# Sexual Dimorphism in the Fly Brain

**DOI:** 10.1016/j.cub.2010.07.045

**Published:** 2010-09-28

**Authors:** Sebastian Cachero, Aaron D. Ostrovsky, Jai Y. Yu, Barry J. Dickson, Gregory S.X.E. Jefferis

**Affiliations:** 1 Division of Neurobiology, MRC Laboratory of Molecular Biology, Cambridge CB2 0QH, UK; 2 Research Institute of Molecular Pathology, Dr. Bohr-Gasse 7, 1030 Vienna, Austria

## Abstract

**Background:**

Sex-specific behavior may originate from differences in brain structure or function. In *Drosophila*, the action of the male-specific isoform of *fruitless* in about 2000 neurons appears to be necessary and sufficient for many aspects of male courtship behavior. Initial work found limited evidence for anatomical dimorphism in these *fru+* neurons. Subsequently, three discrete anatomical differences in central brain *fru+* neurons have been reported, but the global organization of sex differences in wiring is unclear.

**Results:**

A global search for structural differences in the *Drosophila* brain identified large volumetric differences between males and females, mostly in higher brain centers. In parallel, saturating clonal analysis of *fru+* neurons using mosaic analysis with a repressible cell marker identified 62 neuroblast lineages that generate *fru+* neurons in the brain. Coregistering images from male and female brains identified 19 new dimorphisms in males; these are highly concentrated in male-enlarged higher brain centers. Seven dimorphic lineages also had female-specific arbors. In addition, at least 5 of 51 *fru+* lineages in the nerve cord are dimorphic. We use these data to predict >700 potential sites of dimorphic neural connectivity. These are particularly enriched in third-order olfactory neurons of the lateral horn, where we provide strong evidence for dimorphic anatomical connections by labeling partner neurons in different colors in the same brain.

**Conclusion:**

Our analysis reveals substantial differences in wiring and gross anatomy between male and female fly brains. Reciprocal connection differences in the lateral horn offer a plausible explanation for opposing responses to sex pheromones in male and female flies.

## Graphical abstract

**Graphical Abstract:**

**Figure:**
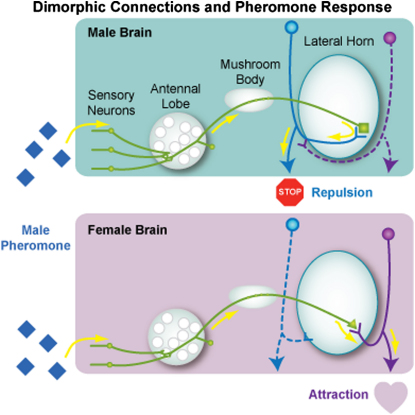

**Highlights:**

► *Drosophila* higher brain centers show robust volume differences between the sexes ► 22 of 62 classes of *fruitless* neurons have sex-specific projections in the brain ► These lead to >700 sites of potentially dimorphic connectivity in the brain ► Anatomical differences in olfactory neurons likely alter pheromone processing

## Introduction

**Figure 1:**
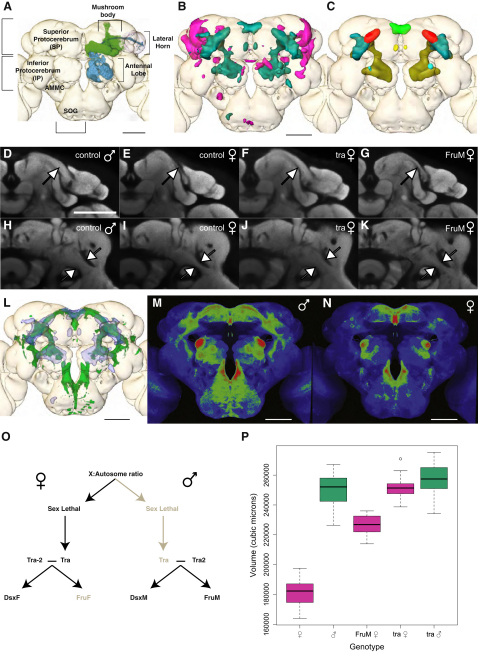
Male-Enlarged Regions Are Genotype Specific and Colocalize with *fruitless* Expression (A) Schematic of *Drosophila* neuropil compartments and olfactory system. Projection neurons are cyan, mushroom body neurons are green. (B) Surface rendering of male (green)- and female (magenta)-enlarged regions on our template brain. (C) Summary of main male-enlarged regions (MERs). Green: pars intercerebralis; red: arch; mustard: ring; turquoise: lateral junction; light blue: DA1 glomerulus; yellow: tips of mushroom body gamma lobes. (D–K) Two individual slices from average template brains for control male (D and H), control female (E and I), *tra* mutant female (F and J), and *fru^M^* mutant female (G and K). In (D)–(G), arrow indicates male-enlarged arch; in (H)–(K), it shows the male expansion of the ring neuropil. (L) Overlap between MERs (blue) and *fru*^Gal4^ expression pattern (green). (M and N) Z projections (standard deviation) of the *fru*^Gal4^ driving CD8-GFP in males (M) and females (N). For clarity, cell bodies in the surface of the brain and the mushroom bodies have been removed. A false color lookup table has been applied to emphasize contrast. (O) Schematic of *Drosophila* sex determination hierarchy. Bold-type gene products are active, gray are inactive. (P) Quantification of the volume of the MER for control and mutant flies. The central line indicates the median, the box represents the 25% and 75% quartiles, and the whiskers extend to ±1.5 times the interquartile range. Scale bar in all panels represents 50 μm. See also Figure S1.

The fruit fly *Drosophila melanogaster* displays robust, highly stereotyped and dimorphic sexual behaviors [1] that provide an ideal model system to study the genetic and neural basis of innate behavior. The genetic pathways that translate chromosomal sex into dimorphic behavior have been studied extensively [2, 3] (Figure 1O). Early studies using sex mosaics mapped different steps of male courtship to broad regions of the central nervous system [4, 5]. Such results suggest that there are anatomical and functional differences between the sexes in these brain regions. At the level of gross anatomy, few structural dimorphisms have been found, and most are small [6, 7]. However, three olfactory glomeruli show volume differences of 25%–60% [8, 9], and two of these have been linked to sex-specific odor processing.

Differences in gross anatomy can identify regions involved in sex-specific behavior, but ultimately we must understand how circuit level anatomy and function differ between the sexes. *fruitless* (*fru*) mutant flies, in which male behavior is selectively disrupted [10, 11], have provided a key entry point to this problem. *fru* is a complex locus with multiple promoters encoding putative transcription factors [12, 13]. The P1 promoter is active in a small fraction of neurons in both sexes and is critical for the sex-specific function of *fru*. In males, default splicing of P1 transcripts results in functional proteins, collectively termed FruM; in females, alternative splicing produces nonfunctional FruF proteins [14, 15]. Behavioral and molecular analysis of *fru* mutants suggests that FruM is required for normal courtship behavior, including inhibition of same-sex courtship [3]. Strikingly, when male splicing is forced, the resultant females court wild-type females [16, 17]. Therefore, the action of FruM on P1-mRNA-expressing neurons (henceforth *fru*+ neurons) appears to be sufficient to determine many aspects of male behavior, and at least some of these neurons must be sexually dimorphic in number, morphology, or function.

One major question in understanding the origin of sex-specific behaviors in general, and the actions of *fru* in particular, is the extent to which they originate from anatomical or functional differences in specific neural circuits. Early anatomical studies that used antibodies against FruM protein [14] counted 1700 *fru+* neurons in 20 groups across the brain and ventral nerve cord (VNC) of males but could reveal little about their anatomy.

The full arborization pattern of all *fru+* neurons was revealed in *fru*^GAL4^ transgenic flies, where a Gal4 insertion targeted to the *fru* locus was used to express GFP [9, 17]. Surprisingly, these studies found no evidence of anatomical differences, although a subsequent study identified differences in the VNC [18]. However, visualizing subsets of *fru+* neurons identified one clear projection dimorphism [19] and two male-specific lineages [20].

It has been proposed that *fru+* neurons form an interconnected circuit [9, 17, 20]. The only clear example of this is in the olfactory system, where both olfactory receptor neurons that detect the male pheromone cVA (a repellent for males and an aphrodisiac for females [21]) and their synaptic partners, the projection neurons (PNs), are *fru+* [9, 22]. However, PN responses to cVA are indistinguishable in males and females, strongly suggesting that sex-specific behavior depends on circuit differences beyond the antennal lobe [22]. Intriguingly, these PNs have a subtle difference in axon terminals in the lateral horn (one higher olfactory center, Figure 1A). However, whether this difference is an anatomical substrate for dimorphic olfactory behavior is unclear, because the identity and structure of the relevant downstream neurons is unknown.

By carrying out a global characterization of *fru+* neurons, we now address three key questions. How many *fru+* neurons are anatomically dimorphic? Where are these dimorphisms located? And how can these dimorphisms alter connectivity and information flow?

## Results

### Large Structural Dimorphisms in Fly Brains

Regional differences in brain anatomy between the sexes can identify the location of circuit level dimorphisms. Previous work examining the whole *Drosophila* brain measured the volumes of predefined regions of interest and found only weak differences [6]. We used deformation-based morphometry [23, 24], which requires no prior hypotheses about the location of volume differences. The method starts with nonrigid registration of many brains onto a template, followed by volume comparisons at every voxel (a pixel in a 3D image).

We analyzed 40 male and 40 female brains and found striking regional volume differences (Figure 1B). We calculated a t statistic for the volume difference at every voxel in the brain and identified male- and female-enlarged regions (t > 5 for an across-brain alpha of 0.05; see Experimental Procedures). Of the total brain neuropil volume, 4.5% and 3.5% were enlarged in males and females, respectively. We then measured the volume of these regions in another group of 80 brains. Male-enlarged regions (MERs) were on average 41.6% larger in males, whereas female-enlarged regions (FERs) were 17.9% larger in females.

The MER includes previously described neuropils: the DA1 glomeruli of the antennal lobe, the pars intercerebralis, and the medial tips of the mushroom body gamma lobes (see Figure 1A for a description of brain regions). However, the strongest statistical signals correspond to three previously unidentified regions that we called the protocerebral arch, ring, and junction (Figure 1C). Visual inspection of the neuropil (as revealed by the presynaptic antibody nc82) showed that the pars intercerebralis and junction (data not shown), arch (Figures 1D and 1E), and ring (Figures 1H and 1I) are substantially enlarged in males. Indeed, these differences were sufficient to determine the gender of brains from unregistered nc82 confocal stacks with 100% accuracy (3 blinded observers, 98 brains each). Similarly, the sex of every registered brain in our data set could be automatically predicted by calculating the volume of the MER. The FERs were less bilaterally symmetric (Figure 1B) and less consistent between the two groups of 80 brains. We carried out a similar volumetric analysis in the VNC, finding substantial male- and female-enlarged regions (see Figure S1 available online).

### *fru+* Neurons and the Male-Enlarged Region

The locations of volumetric differences in the brain are strongly reminiscent of the expression pattern of *fruitless*-Gal4 [9, 17], suggesting that *fru* may help to establish these differences. To confirm the colocalization, we registered brains expressing membrane-targeted GFP driven by *fru*^GAL4^ to our template brain and then overlaid the MERs (Figure 1L). Indeed, the MER was tightly filled by *fru+* processes. However, not all *fru+* processes are in the MER, with notable exceptions including the mushroom bodies and the subesophageal ganglion.

Previous studies reported no overt sexual dimorphisms in the arborizations of all *fru+* neurons. Given the strong overlap between *fru*^GAL4^ expression and the highly dimorphic MER, we revisited this issue. To our surprise, we found that females lack most of the *fru+* processes that are contained within the MER in males (Figures 1M and 1N). We therefore hypothesize that these substantial volumetric differences result from an increase in either the number of *fru+* neurons or their arborizations in these regions.

Two genes, *sex lethal* and *transformer* (*tra*), have the properties of master regulators, specifying both somatic morphology (through *doublesex*) and behavior (*doublesex* and *fru*) [2, 16, 25] (summarized in Figure 1O). *tra* mutant females have male body morphology, and we observed a corresponding complete transformation in their neuropil morphology (Figures 1D, 1F, 1H, and 1J). Furthermore, the MER was not significantly different in size in *tra* females, *tra* males, and control males (Figure 1P), nor are there any other brain regions that are significantly different between *tra* mutant males and females (data not shown). Similarly, VNCs from *tra* mutant males and females had no volumetric differences (Figure S1C).

FruM is required to establish volumetric differences in three antennal lobe glomeruli [9]. Does *fru* influence the morphology of the rest of the brain? Indeed, females expressing the male isoform FruM have male neuropil morphology (Figures 1G and 1K). However, when we quantified the MER volume, we found an incomplete transformation (Figure 1P). Compared with control females, the MER is 38% ± 6% (mean ± standard deviation) enlarged in control males but only 25% ± 5% enlarged in *fru*^M^ females (p = 0.0002; see Experimental Procedures). This mirrors the incomplete behavioral transformation of *fru*^M^ females, which court other females but do not copulate. Full transformation may also require the neuronal actions of *doublesex* [18, 20, 26–28].

### More than 110 Neuroblasts Make *fru+* Neurons

Given the central role of *fruitless* in male behavior, we carried out a comprehensive study of *fru+* neurons, focusing on dimorphic anatomy. Although only 2%–3% of neurons are *fru+*, extensive overlap makes inferences about individual projection patterns difficult (Figures 1M and 1N), so we used mosaic analysis with a repressible cell marker (MARCM) [29] to break *fru+* neurons into smaller groups. The central brain is generated by about 180 neuroblasts (neural stem cells) per hemisphere; each produces a specific group of neurons, usually consisting of only one or two morphological classes [30]. Using MARCM, we generated brains in which only those *fru+* neurons made by one or a few neuroblasts are labeled with a membrane-targeted GFP. From approximately 3000 mosaic brains and 800 VNCs, we obtained, on average, 28 images of each neuroblast clone in the central brain (reaching saturation; see Supplemental Data) and 6 in the VNC (where we have identified most clones). See Figure S2, Table 1, Table S1, and Supplemental Data.

In the central brain, we identified 62 *fru+* neuroblast lineages (including all four mushroom body lineages) containing between 1 and 59 cells (median 23); in the VNC, we identified 51 lineages. Neuroblast clones that contain *fru+* cells also contain *fru*− cells; for instance, the full lateral PN clone aDT-e has 193 cells in total [31], of which 29 are *fru+*. This surprising developmental diversity was paralleled by a diverse set of neuronal morphologies (Figure S2 and Movie S1). By coregistering male and female brains to our template, we directly compared labeled clones from both sexes (see Supplemental Experimental Procedures). This substantially increased the speed and sensitivity of identifying dimorphisms and helped to establish that some highly dimorphic clones are generated by the same neuroblast. Because the cell body position, point of entry of the neurite into the neuropil, and core neurite arbors were always consistent and substantially more similar to each other than any other clone, we concluded that these neurons must be generated by the same neuroblast. The alternative explanation, that a male-specific neuroblast is located at the same position as a female-specific neuroblast that is developmentally unrelated, is more complex but equally interesting.

Of 62 neuroblast lineages, 10 are dimorphic in cell number (p < 0.05 two-tailed t test), with two others approaching statistical significance (Table 1). We count approximately 990 *fru+* cells per central brain hemisphere in the male (excluding mushroom body neurons). The corresponding cell count for *fru*^GAL4^ driving a nuclear marker is 950 [9].

**Figure 2:**
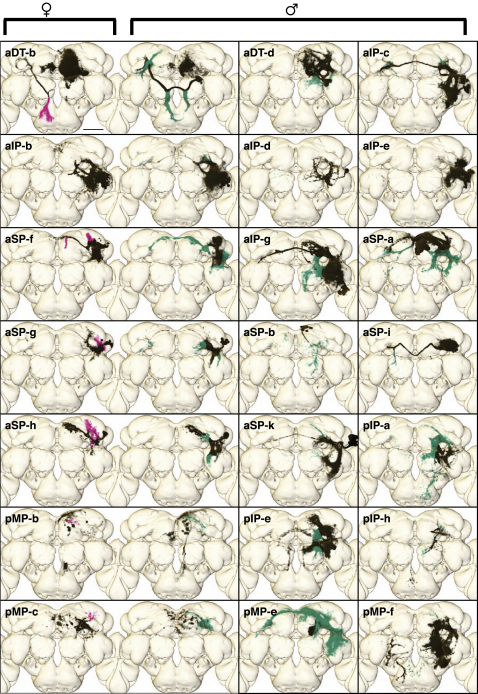
Dimorphisms in *fru+* Neuronal Arbors Volume renderings of representative clones with sexually dimorphic projections. Nondimorphic regions are black, male-specific arbors are green, and female-specific arbors are magenta. Note that some black regions look different between the sexes in these examples, but comparison of multiple examples shows that consistent differences are restricted to green and magenta regions. Clones in the left column have dimorphic arbors in both sexes, and representative examples are shown of each of the male and female brains, whereas clones in the right two columns have only male-specific arbors. In all cases, sex-specific arbors are either missing or much weaker in the other sex. Scale bar represents 50 μm. See also Figure S2 and Movie S1.

We examined the clones for evidence of projection dimorphisms. Three dimorphisms have previously been reported, including the male-specific clone P1 [20] (pMP-e), which extensively innervates the protocerebrum; mAL [19] (aDT-b), which has dimorphic processes in both the protocerebrum and the subesophageal ganglion; and the axon terminals of DA1 olfactory projection neurons [22] (aDT-e). We identified 19 additional clones with dimorphic projections in the central brain (Figure 2), taking the total count to 22. All dimorphic clones have some male-specific processes. Most also have other arbors that are denser in males than females (Figure 2). Finally, seven clones have female-specific processes. Although we did not achieve saturation in the VNC, we nevertheless identified five dimorphic clones (Figure S2).

*fru* and *doublesex* are the only known outputs of the sex-determination pathway in the brain, so *doublesex*-expressing cells may also show dimorphisms. The population of *doublesex* cells is smaller and partially overlapping with the *fru+* population. In the central brain, we observe about 50 *doublesex* cells that are *fru*− (see Supplemental Data), so dimorphisms that we have described in the 990 *fru+* neurons likely represent the large majority of dimorphic anatomy in the fly brain as a whole.

### *fru+* Dimorphic Regions Overlap with the Male-Enlarged Region

**Figure 3:**
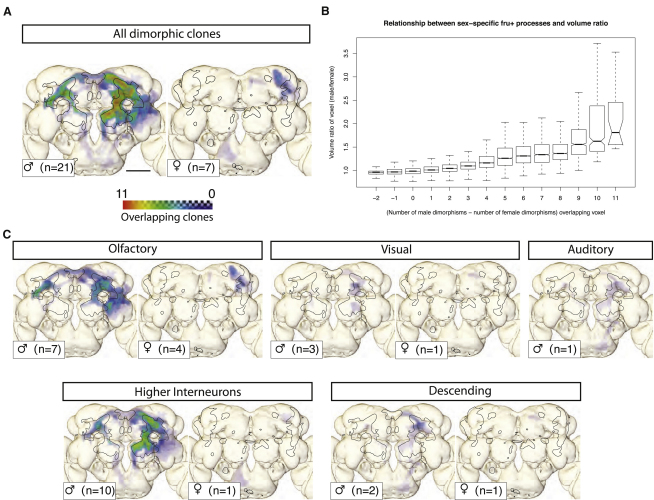
Distribution of *fru+* Dimorphisms in the Brain (A) Dimorphic projections of *fru+* neurons overlap dimorphic neuropil regions. Left shows overlap between male-specific arbors of *fru*+ clones and MER of the neuropil; right shows overlap between female-specific arbors and the female-enlarged regions (FERs) of neuropil. Colored regions are Z projections of the dimorphic arbors shown in Figure 2. Because these images are derived from unilateral mosaic analysis with a repressible cell marker (MARCM) clones with cell bodies on the fly's left (which is on the right of this figure), they are not symmetric. The MER or FER is outlined in each panel as appropriate. Color scale: transparent (no clones) to red (11 clones). Although there are 21 dimorphic clones in males, the region of maximum density corresponds to the overlap of a subset of 11 of those clones. (B) Volumetric dimorphism and sex-specific arbors. There is a strong positive relationship between how many male (or female) specific arbors overlap any given voxel and the male:female volume ratio. For example, voxels with the highest number of overlapping dimorphic arbors (11) also have the highest volume ratio (median 81% male-enlarged). Box plot is as in Figure 1P with the addition of notches to indicate the 95% confidence interval for difference between medians. (C) Clones from (A) broken into functional groups (see Table 1). Each panel contains the dimorphic regions of all clones that belong to a given category. The number of clones in each category that have dimorphic regions and therefore contribute to each panel is shown at the bottom left (e.g., there are 16 visual clones, but only three have male dimorphic processes, and one has female dimorphic processes). Color scale is the same as in (A). Scale bar represents 50 μm.

Having identified the comprehensive set of dimorphisms in *fru+* neurons, we asked where these dimorphisms are located in the brain. We isolated the dimorphic arbors from the 21 clones in Figure 2 and overlaid them on our template brain (Figure 3A). Dimorphic arbors are widespread in males, extending over 11% of the neuropil (see Experimental Procedures) and potentially forming sex-specific connections with many different neurons. In females, dimorphic arbors extend over 2.1% of the brain. Although widely distributed, dimorphic arbors are heavily enriched in specific brain regions.

We found a significant overlap between regions enriched in male-specific arbors and the MER (Figure 3A). This overlap is significantly stronger than the overlap with the whole *fru*^GAL4^ pattern calculated earlier. Furthermore, there is a strong positive relationship between the total number of male-specific processes that overlap a given voxel and the extent to which the voxel is male enlarged (Figure 3B). We also identified a region in the lateral superior protocerebrum enriched in female-specific arbors that overlaps with the FER (Figure 3A).

We then looked in more detail at the distribution of dimorphisms in the *fruitless* circuit. We visualized dimorphic arbors of clones falling into five broad groups (see Table 1 and Figure 3C). We found olfaction to be the sensory modality with the highest number of dimorphic clones (n = 7). Dimorphic processes from male olfactory clones are highly enriched in the ring and the junction. In addition, four out of seven have female-specific processes innervating the lateral superior protocerebrum. In contrast, there are fewer dimorphic clones among the visual (3) and auditory (1) groups.

The largest group, higher interneurons, includes 10 dimorphic clones that we could not directly associate with any sensory system and that do not have descending processes. Given their position within the circuit, these neurons appear likely to integrate information from multiple sensory systems. Their dimorphic arbors are highly enriched in the MER, especially the arch and ring, and, as a result, may have extensive interconnections in males. We identified two descending clones with dimorphic arbors, and both appear to have dendrites in the arch and junction (Figure 3C).

### *fru+* Dimorphisms Can Change Connectivity

**Figure 4:**
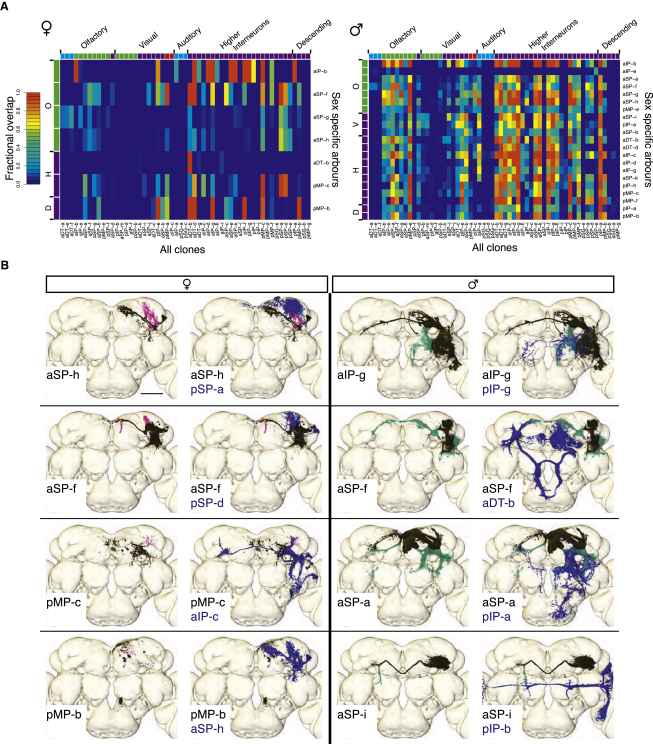
Dimorphic *fru+* Neurons Likely Form Dimorphic Connections (A) Heat maps showing overlap between dimorphic arbors (y axis) from Figure 2 and all clones (x axis). Female-specific arbors are on the left, male-specific arbors are on the right. Red indicates strong overlap, blue indicates no overlap, as per the scale bar. Where two clones have multiple regions of overlap, the strongest overlap is plotted. Clones are grouped by sensory system (see Table 1). Just above and to the left of the heat map are color-coded indicators of the proposed sensory role (i.e., blue: second-order sensory clones, green: third-order sensory clones, purple: higher-order sensory clones, red: visual centrifugal sensory clones). D denotes descending clones, H denotes higher-order clones, V denotes visual clones, and O denotes olfactory clones. (B) Sexually dimorphic clones alter potential connectivity in the *fru+* circuit. Eight examples demonstrate differences in potential connectivity when a clone has a sex-specific arbor (four examples from each sex, females on the left). For each example, the left panel shows a dimorphic clone alone with nondimorphic arbors in black, female-specific arbors in magenta, and male-specific arbors in green. The right panel overlays the second clone in blue. Scale bar represents 50 μm. See also Figure S4 and Movie S2.

Places where dimorphisms change the overlap between *fru+* clones are likely sites of dimorphic connectivity. We analyzed the overlap between dimorphic arbors from the 21 dimorphic clones and all other clones in our data set. This identifies many potential sites where connectivity is different in males and females (Figure 4A). For example, in male brains of the 1218 (21 dimorphic arbors × 58 clones) pairs that we examined, 665 (54.6%) have male-specific overlap (see Experimental Procedures, Figure 4B, and Movie S2). Conversely, females can form connections that are not possible in males. In females, 104 of 399 pairs (26.1%) have female-specific overlap (Figure 4A). These overlap differences include discrete changes that could reroute specific information that needs to be differentially processed in the sexes (e.g., aSP-h, aSP-f, aSP-i), as well as much larger changes that may relate to global control of sex-specific behavior (e.g., aSP-a). Our global analysis excluded ascending connections from the VNC, but visual inspection identified at least one interesting dimorphic overlap. dMS-b has male-specific dendrites in the prothoracic segment of the VNC (that appear to receive male-specific gustatory input) and shows a male-specific overlap with the dendrites of aDT-b (Figures S4B and S4C). This parallels what appears to be a direct gustatory input to these neurons [32].

We omitted neuronal polarity in our analysis, partly because this is sometimes uncertain and partly because cases in which two axons overlap could reflect axo-axonal innervation or a situation in which an unidentified third-party neuron can integrate two signals in one sex but not the other. The pair aSP-i and pIP-b (Figure 4B) likely represents a male-specific convergence of two higher visual pathways. Despite such uncertainties, this constitutes the first attempt to tackle dimorphic connectivity on a whole-circuit scale.

### *fru*+ Higher Olfactory Neurons Are Dimorphic

Given that one-third of dimorphic clones are higher olfactory neurons, we closely examined their organization and possible dimorphic connectivity. Previous studies showed that *fru* is expressed in a subset of olfactory receptor neurons and second-order PNs [9, 17, 22]. We now find that more PNs are *fru+* than initially appreciated, because we identify *fru+* clones from three PN lineages [33–35] (aDT-a, aDT-e, and aDT-f; Figure S2). Nevertheless, the strongest expression was consistently detected in DA1 PNs (aDT-e), with strong expression also seen in VA1lm PNs (aDT-a), both of which have been suggested to detect fly odors [36].

**Figure 5:**
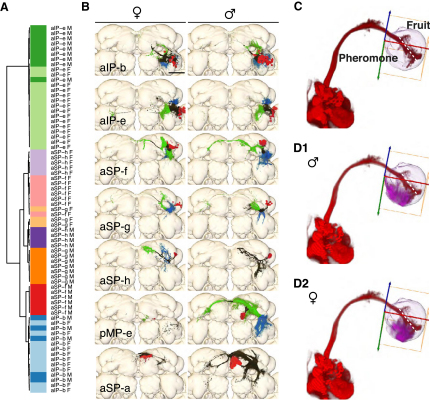
*fru+* Lateral Horn Neurons (A) Cluster analysis of lateral horn innervation patterns of many examples of five classes of *fru+* lateral horn neuron (LHN). Color bars indicate class and sex, with five hues (blue, red, orange, magenta, and green) representing male clones and matched but paler colors representing female clones. For four of these classes, clones of the same sex cocluster but clones of the same class from different sexes do not. The exception is aIP-b, in which male and female samples do not separate (light and dark blue). See Supplemental Experimental Procedures for details. (B) Volume rendering of seven classes of *fru+* LHNs in males and females. Neuronal arbors are colored based on UAS:synaptotagmin staining and neuronal morphology. Cell bodies are in red, axons are in green, dendrites are in blue, unclassified neurites are in black. We did not obtain UAS:synaptotagmin data for clone aSP-a or the male aSP-h clone. (C) The bundle of aDT-a axons (red) traversing the LH serves as a simple landmark to separate pheromone and general odor zones of the lateral horn (dotted white line) [7]. (D1 and D2) Processes of all male (D1) and female (D2) *fru+* LHNs (magenta) are restricted to the pheromone zone. Only LHN neurites within the LH are shown. Scale bar represents 50 μm. See also Figure S3.

Most PN axons make en passant connections in the mushroom body calyx before terminating in the lateral horn (LH). It has previously been shown that many mushroom body neurons are *fru+.* We confirmed this but found no overt anatomical dimorphism in these cells. In the LH, we identified eight *fru+* third-order olfactory neuron lineages. In contrast to the mushroom bodies, seven of eight LH clones have dimorphic projections (Figure 2; Figure 5); three also differ in cell number (Table 1). The sole exception is a pair of local interneurons (pSP-f; see Figure S2). The seven dimorphic clones all have input regions in the LH and output regions in other parts of the brain and can therefore be considered to be principal neurons of the lateral horn (LHNs).

Clones pMP-e and aSP-a are the most dimorphic *fru+* clones in the central brain; their male-specific arbors occupy 6.5% and 2.7% of the central brain neuropil, respectively (Figure 5B). Both clones innervate the LH in males but not females, and both have extensive (and overlapping) arborizations in the MER, suggesting a role as multimodal integrators. The remaining *fru+* LHNs have dendritic arbors that are relatively specific to the LH, occupying from 1% (aIP-b) to 20% (aSP-f). In a cluster analysis, four out of five lineages (aIP-e, aSP-f, aSP-g, and aSP-h) had dimorphic arbors in the LH (Figure 5A), whereas the remaining lineage (aIP-b) occupies the same location in both sexes but is more dense in males. Projection dimorphism was relatively weak in aIP-e, so we confirmed this result by generating a t statistic parametric map of density differences in the lateral horn (data not shown).

**Figure 6:**
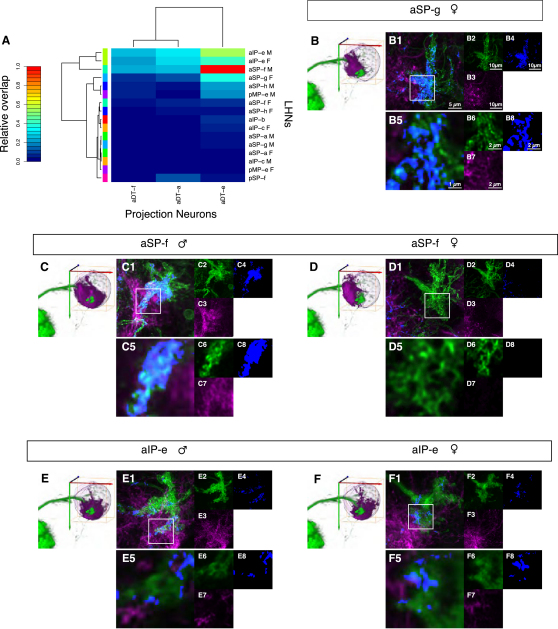
Connectivity in the Olfactory System (A) Heat map representation of registration-based overlap between projection neuron (PN) axons and neurites of all *fru+* LHNs. Color indicates relative overlap score (see Supplemental Experimental Procedures) ranging from 0 (no overlap) to 1 (the strongest observed overlap). (B–B8) Lateral horn from a female with LHN clone aSP-g and PN clone aDT-e, labeled in different colors in the same brain by means of twin-spot MARCM. (B1)–(B4) are Z projections (maximum intensity) of the axon terminals of pheromone-responsive PNs and their potential LHN partners. (B1) shows PNs in green and LHNs in magenta overlaid with a blue channel that indicates places where PN and LHN processes overlap or occupy neighboring pixels (see Supplemental Experimental Procedures). (B2)–(B4) are the individual channels. (B5)–(B8) are a single slice of expanded views of the white box from (B1). Note that in females there are large regions of proximity between the two clones. For orientation, a volume rendering of aDT-e (green) and the LHN clone in the LH is shown to the left of (B1). (C–C8) Twin-spot MARCM lateral horn from a male with LHN clone aSP-f and PN clone aDT-e in different colors. Colors and scale are as in (B). There are extensive regions of proximity between the PNs and the LHNs. (D–D8) Female LHN clone aSP-f and PN clone aDT-e in different colors. There are no regions of proximity around the pheromone-sensitive PN axon terminals, a marked difference from (C). (E–E8) Male LHN clone aIP-e and PN clone aDT-e in different colors. There are limited regions of proximity between the two clones on an order similar to the female in (F). (F–F8) Female LHN clone aIP-e and PN clone aDT-e in different colors. There are limited regions of proximity between the two clones on an order similar to the male in (E).

Having shown that LHNs have dimorphic arbors in the lateral horn, we next evaluated the effect that those dimorphisms might have on connectivity. First, we used registration data to carry out a quantitative analysis of overlap in the LH between *fru+* PNs and LHNs (Experimental Procedures). About one-third of combinations had significant overlap, but certain neurons appear much more likely to form connections (Figure 6A and Figure S5). Some overlaps are sex-specific. For example, aSP-f and aSP-h have the potential to form synapses with aDT-e and aDT-a only in males, and aSP-g can only form synapses with aDT-e, aDT-a, and aDT-f in females.

The combination of image registration error and biological variability results in an accuracy of 2–3 μm [7], limiting the predictive strength of registration-based overlap analysis. To make stronger predictions about connectivity, we used twin-spot MARCM [37] to generate brains with putative pre- and postsynaptic *fru+* clones (Figure 6) labeled with either GFP or RFP. In this way, we obtained a small fraction of brains that had an aDT-e (DA1 PN) clone and an LHN clone marked in different colors. High-resolution confocal microscopy and image deconvolution allowed us to assess the overlap between axons and dendrites with an accuracy close to the optical diffraction limit (<300 nm). We quantified this overlap by measuring what fraction of the PN terminals was within 1 voxel (170 nm) of an LHN arbor. In males, aSP-f dendrites had 32.6% overlap, compared with 0.1% in females (Figures 6B and 6C, respectively). Conversely, aSP-g shows 22.2% overlap in females (Figure 6B); we could not find a useful twin-spot sample in males, but registration-based analysis indicates limited overlap. Interestingly, aIP-e clones, which have strong overlap with DA1 PNs using the registration-based analysis, have only weakly overlapping neurites in twin-spot samples (2.2% and 2.1% in males and females, Figures 6E and 6F, respectively). Taken together, these results validate the registration-based overlap approach for analyzing large data sets while highlighting the need for higher-resolution follow-up to draw firm conclusions.

Previous work identified a 5–10 μm male-specific ventral extension of the axon terminals of pheromone-responsive DA1 PNs in the LH but did not identify their postsynaptic partners [22]. We have now identified all *fru+* LHNs and provide the first direct evidence for dimorphic anatomical connectivity between DA1 PNs and higher olfactory neurons; this difference is a very strong candidate to explain the different behavioral responses of males and females to cVA.

We next looked at *fru+* LHN output. LHNs have distinct axon arbors across the protocerebrum, but all target the protocerebral MERs in males. Furthermore, dendritic dimorphisms within the LH are complemented by strong axonal dimorphisms outside the LH (Figure 5B and Figure S3). In particular, clones aSP-a, aSP-f, aSP-h, and pMP-e have extensive arborizations in the MERs that are absent from females. Conversely, aSP-f, aSP-g, and aSP-h all have female-specific processes that localize to the FER in the dorsal protocerebrum. This topology suggests the presence of a global developmental switch in the output of the pheromone-processing center in the LH between the sexes, with information flow out of the LH being different in males and females.

The MER is so rich in different *fru+* processes that we cannot make strong predictions about which neurons are the most likely postsynaptic partners. However, LHN axon arbors do overlap with one class of descending neuron, pIP-a (Figure S4). These neurons have dendrites that are widely distributed through all regions of the MER and axons that project in a bundle to the VNC, where they have terminal arbors in all three thoracic ganglia (Figure S4A). Thus, olfactory receptor neurons may be separated by only three synapses (antennal lobe, LH, MER) from the VNC. pIP-a probably does not contact motorneurons directly ([38], this issue of *Current Biology*), so it is likely that a minimum of four synapses is required for the olfactory sensory-motor loop.

## Discussion

We carried out the first global, voxelwise analysis of structural differences in an insect brain. This allowed us to identify extensive sex-specific volume differences missed in earlier studies of *Drosophila*. These are so reliable that brain shape can identify the sex of individual animals, which can be of experimental benefit. The strong overlap of these volumetric differences with dimorphic arbors of *fru+* neurons suggests a role in male behavior. Early studies of sex mosaics showed that the superior protocerebrum is a key regulator of courtship behavior [25]. Our results strongly support that localization and identify specific regions in the superior protocerebrum likely to regulate both male and female behavior.

At the circuit level, we have built a 3D atlas containing 62 *fru+* neuroblast lineages in the central brain and 51 in the VNC. These numbers reveal an impressive heterogeneity in the developmental origin of *fru+* neurons. The central brain consists of the cerebrum (made by 100 neuroblasts, 57 of which are *fru+*) and the smaller subesophageal ganglion (80 neuroblasts, 5 of which are *fru+*) [30]. Few of these lineages, or their component neurons, have previously been described in adult flies, making this study a substantial contribution to understanding the development and neuroanatomy of the fly brain.

In contrast to the preliminary conclusion that the *fruitless* circuit is anatomically largely isomorphic [9, 17], we show that one-third of *fru+* neuronal clones are dimorphic. Previous work on sexual dimorphisms in insects has focused on changes that increase the sensitivity of sensory systems to visual [39] or olfactory [40] cues from conspecifics. However, we find that most dimorphisms are in the arbors of *fru+* higher interneurons of the protocerebrum. In those cases where arbors are denser in males than females but still similarly located, the effect could be to increase the strength of connections between the same groups of neurons; this could be analogous to the increases in sensory sensitivity mentioned above, increasing the gain for some signals without fundamentally altering circuit logic. However, all 21 dimorphic clones described in Figure 2 have arbors in locations where there are no processes in the opposite sex; in these cases, it seems very likely that different groups of neurons are connected in the two sexes. The sex-specific projection differences generated during development may therefore be somewhat analogous to the jumper switches in an electronic circuit—a few specific locations where connections can be made or altered in order to change the logic of a largely conserved circuit. Pursuing this analogy, engineers place jumper switches at key locations in order to efficiently alter circuit logic. Can we propose any logic to rationalize the location of *fru+* projection dimorphisms?

The olfactory system provides an excellent example to explore how these circuit level dimorphisms may regulate dimorphic behavior. In the periphery, *fru+* olfactory sensory neurons are more numerous in males [9]. This could enhance sensory sensitivity but cannot explain, for example, why the male pheromone cVA should be repulsive for males but stimulatory for females [21]. We propose that changes in connectivity between second- and third-order olfactory neurons, largely because of shifts in the dendritic arbors of *fru+* lateral horn neurons, result in the rewiring of sensory signals detected by both sexes. We have identified several populations of lateral horn neurons that show precise overlap with the axons of cVA-responsive PNs. This includes two populations with overlap only in males and another with female-specific overlap. We therefore hypothesize that these lateral horn neurons are the first elements in the olfactory pathway that show different responses to the same stimulus in males and females.

In flies, odors are represented in the first two stages of the olfactory system by the activity of about 50 channels (corresponding to the glomeruli of the antennal lobe). Odors usually activate many channels, and different odors can activate the same channel. PNs show little integration of information across olfactory channels, in clear contrast to third-order neurons [41]. Although cVA is a rather specific signal known to activate only two channels [21, 36], it occurs in a variety of behavioral contexts [42]. Third-order neurons that integrate cVA signals with olfactory signals from other PNs may therefore respond with increased selectivity to behaviorally relevant odor objects such as male and female flies. It would make sense to restrict substantial anatomical dimorphism to such neurons capable of selective integration. More generally, we hypothesize that it would be efficient to alter connections involving higher-order neurons that robustly represent a relevant feature of an external stimulus (e.g., the presence of a fly of the opposite sex) rather than more peripheral neurons, which can be activated by more diverse stimuli.

Outside the olfactory system, a number of relatively discrete dimorphisms could alter the processing of external stimuli or the behaviors to which they are eventually connected. However, the analogy with an electronic circuit that can be (developmentally) reconfigured by altering some connections between the same set of elements is incomplete. There are some dimorphic clones that have many more neurons in males, most obviously pMP-e and aSP-a, which have extensive male-specific processes in the MER. The MER is rich in dimorphic *fru+* neurons and appears to receive diverse sensory information and to be connected to the VNC (Figure 2; Figure 3; Figure S4) [38]. Masculinization of pMP-e (P1) in otherwise female flies is strongly correlated with induction of male courtship behavior [20], so it is proposed to initiate male behavior. Although the circuit logic of the MER is not yet clear, we propose two specific models for these highly dimorphic neurons. First, they may integrate multiple sensory streams and, when activated, directly excite descending neurons that control male motor programs. Alternatively, they may reflect the state of excitation of the male by generating a long-lasting potentiation of connections in the MER between sensory inputs specific to particular descending neurons.

Alterations in the presence and wiring of specific circuit elements according to the logic we have just described could result in sex-specific activation of motor programs that are largely conserved between the sexes. This is consistent with the finding that direct activation of *fru+* neurons in the VNC of females can induce courtship song [43]. However, song is abnormal unless those *fru+* neurons are masculinized by *fru*^M^, so there may be similar developmental alterations in motor circuits of the VNC (e.g., Figure S2D). The logic of sexually dimorphic behavior has strong parallels in mice, in which removal of the pheromone-sensing vomeronasal organ from females results in male behavior [44]. The resultant hypothesis proposes that circuits for male and female behavior exist in females but that sensory information from the vomeronasal organ normally represses male behavior. This, then, begs the question of what circuit difference alters the behavioral significance of vomeronasal organ activation. Genetic approaches [45] to identifying and characterizing circuit level differences in mice will find answers in the long run. However, we are now ready to test how dimorphic anatomy can alter neural processing and behavior in *Drosophila*.

## Experimental Procedures

### Fly Strains

*fru+* cells were labeled using the *fru*^GAL4^ line, in which the yeast transcriptional regulator Gal4 has been targeted to the start of the coding sequence for the FruM isoform [9]. The full *fru*^GAL4^ pattern was imaged in flies of genotype *+/(+ or Y); FRT G13 UAS-mCD8-GFP/+; fru*^GAL4^*/+*. Males and females were stained in the same tube and were mounted and imaged together using identical laser power and gain settings, and brain sex was established later by inspection of the MER on the nc82 staining. MARCM labeling used flies of the genotype *y w hs-FLP UAS-mCD8-GFP/(+* or *Y); UAS-mCD8-GFP FRT*^G13^*/FRT*^G13^ *tubP-GAL80; fru*^GAL4^*/+.* We initially analyzed the distribution of presynaptic compartments in female *fru+* clones using *y w hs-FLP UAS-mCD8-GFP/UAS-Syt-HA; UAS-mCD8-GFP FRT*^G13^*/FRT*^G13^ *tubP-GAL80; fru*^GAL4^*/+* flies. We then extended the analysis to both sexes using *UAS-Syt-HA/(+ or Y); UAS-mCD8-GFP FRT*^G13^*/FRT*^G13^ *tubP-GAL80; fru*^GAL4^*/MKRS hs-FLP* flies. In all cases, MARCM clones were generated by heat shocking first instar larvae for 17 min (males) and 23 min (females) at 37°C between 0 and 3 hr after larval hatching. For deformation-based morphometry analysis, we used MARCM flies as the control group, although only individuals without the MKRS chromosome were used. *tra* mutant females were *tra*^1^*/Df(3L)st-J7*, whereas *tra* mutant males were *w*^ap^*; tra*^1^*/Df(3L)st-J7*. *fru*^M^ mutants [16] were *+/+;;fru*^M^*/+.* Twin-spot MARCM [37] was carried out by crossing *hsFlp [**1**]; FRT40A,UAS—Cd2::rfp,UAS—gfp-Mir* females to *yw; FRT40A,UAS-mCD8::gfp,UAS-rCD2-Mir/CyO; fru^GAL4^/TM3,Sb* males. These twin-spot MARCM progeny were collected as newly hatched larvae and heat shocked between 30 min and 1 hr.

### Immunochemistry

Fixation and immunochemistry were carried out as described by [7], except that the blocking step was overnight at 4°C. Primary: mouse anti-nc82 [46] 1:20–1:40, rat anti-CD8a (Caltag, Burlingame) 1:100, chicken anti-GFP (Abcam, ab13970) 1:1000, rat anti-HA (Roche, 11 867 423 001) 1:200, mouse anti-rCD2 (Abcam, ab33833), affinity-purified rat polyclonal anti-Doublesex (kind gift, M. Arbeitman) 1:200. Secondary: Alexa-568 anti-mouse (Invitrogen) 1:200, Alexa-488 anti-rat (Invitrogen) 1:200, Alexa-488 anti-chicken (Invitrogen) 1:200, Alexa-633 anti-rat (Invitrogen) 1:400. Prolonged incubation (2–3 days at 4°C) with primary and secondary antibodies was required for homogeneous staining. Specimens were whole mounted in Vectashield (Vector Labs) on charged slides to avoid movement.

### Image Acquisition

Confocal stacks were acquired using a Zeiss 710 confocal microscope equipped with a motorized stage, which allowed unattended imaging of multiple samples. Brains were imaged at 768 × 768 pixel resolution every 1 μm (0.46 × 0.46 × 1 μm) using an EC Plan-Neofluar 40×/1.30 oil objective and 0.6 zoom factor. VNCs were imaged at 1024 × 768 every 1 μm (0.47 × 0.47 × 1 μm) using an LD LCI Plan-Apochromat 25×/0.8 objective with immersion oil. Twin-spot MARCM images taken with a Plan-Apochromat 63×/1.4 oil objective at 3× zoom contained about 300 768 × 768 pixel slices, with a voxel size of 0.06 × 0.06 × 0.15 μm. All images were taken using 16-bit color depths.

### Image Processing and Analysis

Image registration and deformation-based morphometry were carried out largely as described [7]. As before, nonrigid registration was based on confocal images in which the neuropil is stained with the nc82 antibody. We constructed new shape-average templates for the brain and VNC using nc82 confocal stacks, including an average male and an average female template brain. Because we found large differences in morphology between male and female brains, we computed a new template by shape-averaging the male and female templates. We refer to this as an intersex template, and it is used in all figures unless indicated otherwise. To aid visualization and subsequent analysis, we extracted *fru+* clones from raw image stacks by multiplying by a standard binary mask for each lineage. Full details and all relevant source code are available in the Supplemental Experimental Procedures and web links therein. In addition, single example image stacks for all *fru+* clones in this manuscript, as well as template brains and relevant software tools, can be downloaded from http://flybrain.stanford.edu. Finally, all raw and processed brain images are freely available from the authors.

## Figures and Tables

**Table 1:** Summary of *fru+* Clones

Clone Name	Cell Number	SD (Males)	SD (Females)	Dimorphic Projections
**Olfactory Clones**				

*Sensory Neurons*				
SN-e	NA	NA	NA	−
*Second-Order Neurons*				
aDT-a	32.8	6.40	5.52	−
aDT-e	29.0	7.96	6.50	♂
aDT-f	21.0	8.49	5.79	−
*Third-Order Neurons*				
aIP-b	32.5 (19.6)	5.57	5.64	♂♀
aIP-e	27.0	4.24	2.17	♂
aSP-a	53.0 (22.6)	1.22	1.82	♂
aSP-f	23.2 (18.6)	2.59	5.03	♂♀
aSP-g	13.4	0.89	4.97	♂♀
aSP-h	5.0	0.82	0.45	♂♀
MB	NA	NA	NA	−
pMP-e	38.4 (9)	16.32	1.41	♂
pSP-f	2.0	0	0	−
*Higher-Order Neurons*				
aSP-d	48.3	8.69	4.77	−

**Visual Clones**				

*Second-Order Neurons*				
M	NA	NA	NA	♂
*Third-Order Neurons*				
Lo	NA	NA	NA	−
pIP-b	24.0	4.24	4.21	−
aSP-m	6.0	4.12	3.46	−
pIP-d	NA	NA	NA	−
aIP-f	23.0	1.41	7.97	−
pSP-b	31.0 (19)	4.85	2.92	−
pMP-b	22.0	4.42	3.35	♂♀
*Higher-Order Neurons*				
aDT-h	51.0	14.02	10.20	−
aSP-i	21.8	4.03	1.64	♂
aSP-j	25.8	6.46	NA	−
aDT-c	1.0	0	0	−
pIP-e	34.0	9.90	2.83	♂
aSP-b	15.8 (10.2)	1.79	4.77	♂
*Centrifugal Neurons*				
pMP-a	33.5 (23.2^∗^)	7.94	8.04	−
pIP-c	13.8	4.27	3.87	−

**Gustatory Clones**				

SN-a	NA	NA	NA	−
SN-b	NA	NA	NA	−
SN-c	NA	NA	NA	−

**Auditory Clones**				

*Sensory Neurons*				
SN-d	NA	NA	NA	−
*Second-Order Neurons*				
aDT-e	29.0	7.96	6.50	♂
aIP-a	22.7	1.53	6.52	−
aSG-a	10.5	3.32	2.22	−
pMP-g	1.0	NA	NA	−
pSG-a	5.5	0.58	1.71	−
pSG-d	8.0	1.41	5.25	−
pIP-a	14.3 (6.4^∗^)	NA	1.52	♂
pSG-b	12.0	5.60	2.75	−
pSG-c	NA	NA	NA	−
*Higher Interneurons*				
aDT-b	58.6 (28.6)	10.31	8.02	♂♀
aDT-d	25.2	3.27	3.85	♂
aDT-g	22.4	2.30	6.24	−
aIP-c	30.8 (21.4)	1.30	3.71	♂
aIP-d	1.0	0	0	♂
aIP-g	32.0	10.03	4.62	♂
aIP-h	11.0	4.24	4.04	−
aSP-c	3.0	1.41	0.00	−
aSP-e	1.0	NA	4.77	−
aSP-k	29.2 (20.2)	3.27	3.49	♂
aSP-l	24.0	0.96	1.10	−
pIP-f	1.0	0	0	−
pIP-g	6.2	2.28	2.68	−
pIP-h	1.0	0	0	♂
pIP-i	1.0	0	0	−
pIP-j	2.0	NA	NA	−
pMP-c	11.8	3.96	3.54	♂♀
pMP-d	1.0	0	0	−
pMP-f	21.3	3.21	7.30	♂
pSG-e	1.0	0	0	−
pSP-a	6.4	1.67	NA	−
pSP-c	1.0	0	0	−
pSP-d	NA	NA	NA	−
pSP-e	2.5	0.71	0.96	−

**Descending Clones**				

aSG-a	10.5	3.32	2.22	−
pIP-a	14.3 (6.4^∗^)	3.06	1.52	♂
pMP-b	22.0	4.42	3.35	♂♀
pMP-g	NA	NA	NA	−
pSG-b	12.0	5.60	2.75	−

Information about clone names, number of cells, and projection dimorphism. Clones are organized into groups based on likely sensory input. Numbers in parentheses indicate female cell number when they are significantly different from males (p < 0.05). ^∗^ indicates a trend toward significance (0.1 > p > 0.05). ♂ indicates male-specific arbors, and ♂♀ indicates male- and female-specific arbors. NA denotes not available. Clones in bold have arbors in the MER. Note that clone aDT-e (lPNs) is both an olfactory clone and an auditory clone in this scheme and that the descending clones are likewise accounted for in other sensory systems. See also Tables S1 and S2.

## Supplemental Information

Document S1. Supplemental Experimental Procedures, Six Figures, and Two Tables

Movie S1. Atlas of Central Brain Clones Projections of two examples of each central brain clone from males and females (see also Figure 2). Cases for which we did not have images with fully isolated clones are not shown.

Movie S2. fru+ Neurons Likely Form Sexually Dimorphic Connections 3D volume renderings showing the dimorphism in the overlap between clones pIP-b and aSP-i (see also Figure 4). aSP-i is shown in black, and its dimorphic arbors are shown in green. The overlapping pIP-b clone is shown in blue.

Movie S3. Registration-Based Overlap of fru+ Projection Neurons and Dimorphic Lateral Horn Neurons 3D volume renderings showing the overlap of the dimorphic LHN clone aSP-f with aDT-e projection neurons (red). Although the male clone (green) has a strong overlap, the female one (magenta) overlaps poorly in the lateral horn (see also Figure 6). The clones in Movie S2 and Movie S3 were obtained from different animals and registered to our intersex template.

Movie S4. Two-Color Labeling Confirms the Absence of Male-Specific Contacts between fru+ Olfactory Neurons in the Lateral Horn of a Female Fly Confocal microscope stacks showing a high-magnification view of the lateral horn (as in Figure 6) from a female. The axonal terminals of aDT-e PNs (green) have very limited overlap with the dendrites of aSP-f (magenta) in females (and extensive overlap in males, Movie S5). Shown in blue are the voxels where there are PN processes less than 1 voxel away from LHN processes. Clones were labeled in different colors (RFP, GFP) in the same animal using twin-spot MARCM.

Movie S5. Two-Color Labeling Shows Male-Specific Contacts between fru+ Olfactory Neurons in the Lateral Horn Confocal microscope stacks showing a high-magnification view of the lateral horn (as in Figure 6) from a male. The axonal terminals of aDT-e PNs (green) have extensive overlap with the dendrites of aSP-f (magenta) in males (but very limited overlap in females, Movie S4). Shown in blue are the voxels where there are PN processes less than 1 voxel away from LHN processes. Clones were labeled in different colors (RFP, GFP) in the same animal using twin-spot MARCM.
