# Supplementary material for: Sexual Dimorphism in the Fly Brain
Source: Curr Biol. 2010 Sep 28;20(18-4):1589–601. doi: 10.1016/j.cub.2010.07.045 (PMC2957842; doi:10.1016/j.cub.2010.07.045)
Supplement: Document S1. Supplemental Experimental Procedures, Six Figures, and Two Tables [file mmc1.pdf]

Current Biology, Volume *20*

Supplemental Information

Sexual Dimorphism in the Fly Brain

Sebastian Cachero, Aaron D. Ostrovsky, Jai Y. Yu, Barry J. Dickson, and Gregory S.X.E. Jefferis

Supplemental Data

Additional Volumetric Analysis

In the central brain the largest contiguous Female Enlarged Regions are in the lateral superior protocerebrum, but we could not see a corresponding difference in nc82 stained neuropil. A second smaller, but significant ($t > 10$), FER is in the dorsal and medial most segment of the ventrolateral protocerebrum and appears to be contiguous with the neuropil in the mushroom body pedunculus; this FER is observable in the nc82 stainings and can be used to differentiate females from males with high accuracy.

Sex specific regions could also be observed in the ventral nerve cord. The largest FER covered a superficial layer of the abdominal ganglion that may relate to female specialisations for oviposition. A second region runs along the most lateral part of the wing neuropil of the mesothoracic ganglion. The male-enlarged region had more complex morphology restricted to the interior of the neuropil, located primarily in the leg neuropil of the prothoracic ganglion and the wing neuropil of the mesothoracic ganglion. This may relate to the sensory role of the forelegs during the tapping stage of courtship and wing circuits regulating courtship song [1–3].

Overlap of *fru*+ Neurons and MER

In males, the highest intensity *fru*+ processes occupy 6.5% of the neuropil while the Male Enlarged Regions occupy 4.5% with 1.7% of neuropil voxels present in both groups; random overlap would predict only 0.29% of neuropil voxels would be double positive. There is a highly significant association between these two groups of voxels (Fisher's exact test, odds ratio 11.4, $p<2.2e-16$). We calculated the overlap between voxels containing at least two male-specific arbours (6.7% of the neuropil) and the MER (4.5% of the neuropil), finding that double positive voxels occupied 2.7% of the neuropil (odds ratio 19.9, $p<2.2e-16$, Fisher's exact test). Similarly in females, the overlap between voxels containing at least two *fru*+ female-specific arbours (0.8% of the neuropil) and the female-enlarged region (3.5%) identifies double positive voxels occupying 0.12% of the neuropil. Although a relatively small overlap, this is still significantly above chance (odds ratio 5.1, $p<2.2e-16$, Fisher's exact test).

Nomenclature of *fru*+ Clones

While there are other schemes that have been in use for naming fruitless clusters [4], these groupings were inevitably based on somewhat arbitrary cell groups, since nothing was known

about the projection patterns or developmental origin of the neurons in question. We have coordinated a new scheme with [5] where the fly's brain is divided into the deutocerebrum (DT), the anterior and posterior superior protocerebrum (aSP and pSP respectively), the posterior medial protocerebrum (pMP), the anterior and posterior inferior protocerebrum (aIP and pIP respectively), the anterior and posterior suboesophageal ganglia (aSG and pSG respectively), and the mushroom body (MB) (see Figure 1A). Clones are placed in a region depending on the location of the cell bodies and primary arborisation, the great commissure is used as the dividing line between the two (adapted from [6]). Where this is ambiguous the area of primary innervation is used to select a position along the A—P axis. After all clones had been identified, each clone received a unique lower case letter appended to the name stem; the first clone was the one closest to the midline in the dorsal-most part of the region. Starting with the first clone the next clone clockwise when looking at frontal sections of the brain was given the next letter and so on. See Table S2 on page 39 for correspondences between names of *fru* clusters from different studies.

Naming in the VNC was likewise changed from previous schemes; cell body position dictated the position along dorsal-ventral axis and the region of primary innervation dictated the stem of the name (i.e. pro- , meso-, or metathoracic, Pr, Ms, and Mt respectively). The first clone of a region was the one with cell bodies closest to the midline and most anterior, the next was the one clockwise when looking at a VNC with the ventral side facing the observer and anterior 'up'. The names for the *fru+* regions were suggested in our companion manuscript [5] and adapted in this study based on correspondences with the male-enlarged neuropil regions.

Relationship of This Study to Other Studies of *fru*+ Neurons

Two previous studies applied MARCM to NP21, a GAL4 enhancer trap inserted at the *fru* locus [7, 8]. They described 38 NP21 clones in the central brain including one dimorphic and two male-specific clones. 26 of those 38 clones (353 neurons) have a homologue in our dataset; we cannot match the remaining 12 clones (45 neurons). See Table S2 on page 39 for detailed cross-referencing of our data against these earlier studies. In comparison with those studies, we describe the anatomy of 85 additional *fru+* clones across the brain and VNC. All clones are registered in the same reference space, allowing us to compare male and female anatomy for all brain and most VNC clones. We identify 24 new dimorphisms. We also describe the complete arborisations of 13 clones that connect the brain and VNC. Finally all of our 3D data are freely available.

Table S2 also details the correspondence between our 62 central brain neuroblast MARCM clones and the 60 clusters of [5] derived from an intersectional scheme using a fru^{Flp} allele. Most large groups of neurons could be clearly identified in the two datasets, but a significant number of our clones remained without a corresponding identification. Some fru^{Flp} clusters corresponded to as many as 3 MARCM clones (aDT3 vs aDT-a, aDT-e, aDT-f). Conversely clone pIP-c corresponds to cluster pIP3 and pIP7; these are two groups of neurons with distinct morphologies that are generated by the same neuroblast. Detailed analysis of the raw data using computerised pattern recognition may be the best means of finding all correspondences (Nicolas Masse, SC, AO, GSXEJ, unpublished observations). We expect that there will be some neurons present in only one data set. The fru^{Flp} approach is limited by the availability of Gal4 lines to generate

intersectional patterns that are sufficiently simple to visualise and distinguish every neuronal class. The *fru*^{GAL4} MARCM approach is more comprehensive in principle because it is possible to label all *fru*+ neurons in small reproducible groups (the neuroblast clones). However we chose to focus on postembryonic neurons by heat shocking experimental animals at larval hatching. Embryonic neuroblast clones were generated only sporadically in our data set, meaning that we likely missed some *fru*+ neurons that are born during embryonic development. For the 62 lineages in our central brain dataset we found a minimum of 3 examples of every clone (28 on average). Although there are differences in the likelihood that different neuroblasts generate MARCM clones, we expect that we have identified all postembryonic *fru*+ lineages. In the VNC there were multiple cases where we obtained only one or two examples of a given clone, so we cannot presume to have identified all possible lineages.

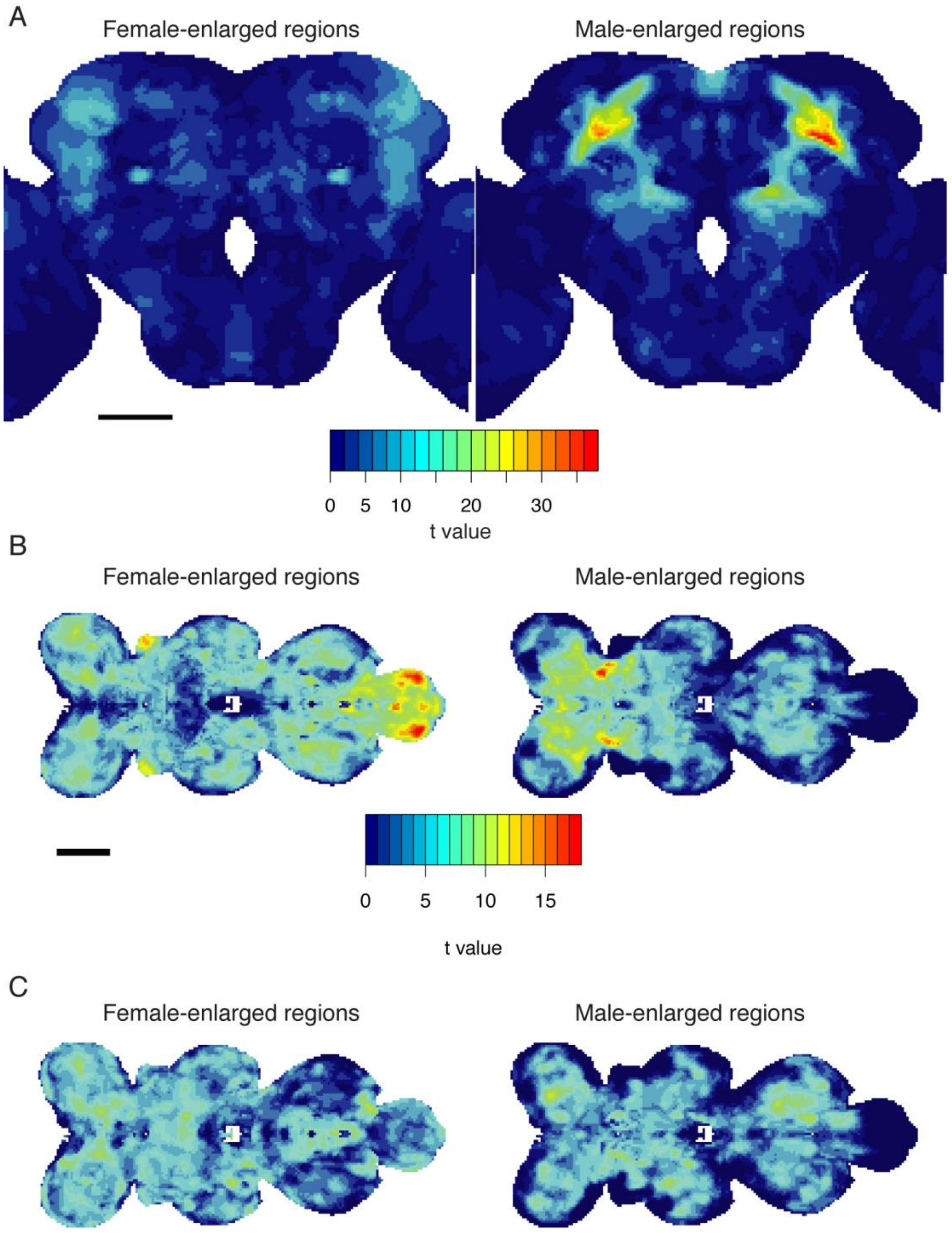

Figure S1. Volumetric Dimorphism (See Figure 1)
t-statistic parametric map of female-enlarged and male-enlarged regions, respectively. The original 3D data have been maximum intensity projected along the z axis. Scale bar = 50µm.
(A) Brain from control animals. Heatmap indicates t-statistic values and is used for both male and female brains.
(B) VNC from control animals. Heatmap differs from (A) and is used in (B) and (C).
(C) VNC from *tra* mutant animals.

A

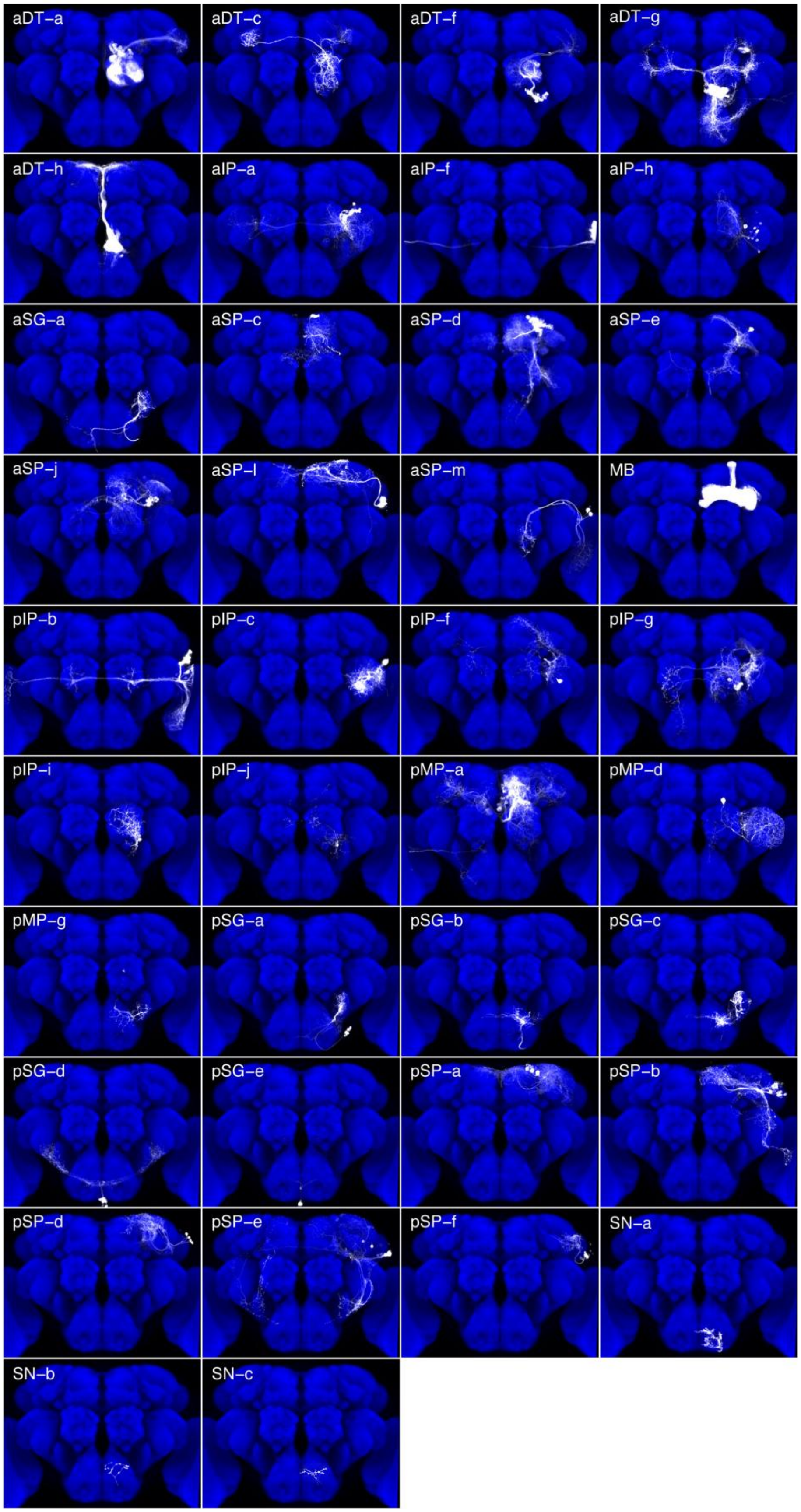

aDT–a
aDT–c
aDT–f
aDT–g
aDT–h
aIP–a
aIP–f
aIP–h
aSG–a
aSP–c
aSP–d
aSP–e
aSP–j
aSP–l
aSP–m
MB
pIP–b
pIP–c
pIP–f
pIP–g
pIP–i
pIP–j
pMP–a
pMP–d
pMP–g
pSG–a
pSG–b
pSG–c
pSG–d
pSG–e
pSP–a
pSP–b
pSP–d
pSP–e
pSP–f
SN–a
SN–b
SN–c

B

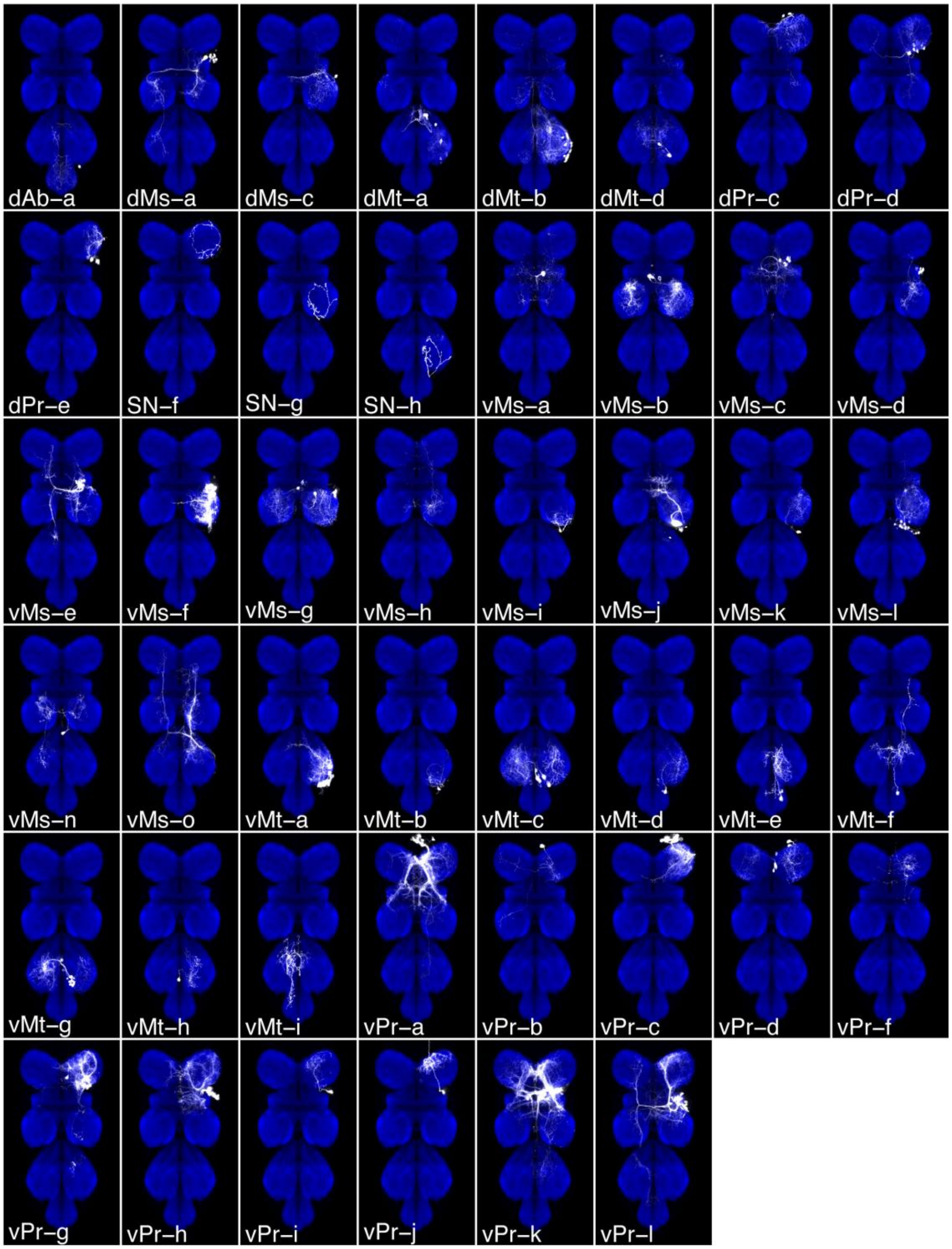
dAb–a
dMs–a
dMs–c
dMt–a
dMt–b
dMt–d
dPr–c
dPr–d
dPr–e
SN–f
SN–g
SN–h
vMs–a
vMs–b
vMs–c
vMs–d
vMs–e
vMs–f
vMs–g
vMs–h
vMs–i
vMs–j
vMs–k
vMs–l
vMs–n
vMs–o
vMt–a
vMt–b
vMt–c
vMt–d
vMt–e
vMt–f
vMt–g
vMt–h
vMt–i
vPr–a
vPr–b
vPr–c
vPr–d
vPr–f
vPr–g
vPr–h
vPr–i
vPr–j
vPr–k
vPr–l

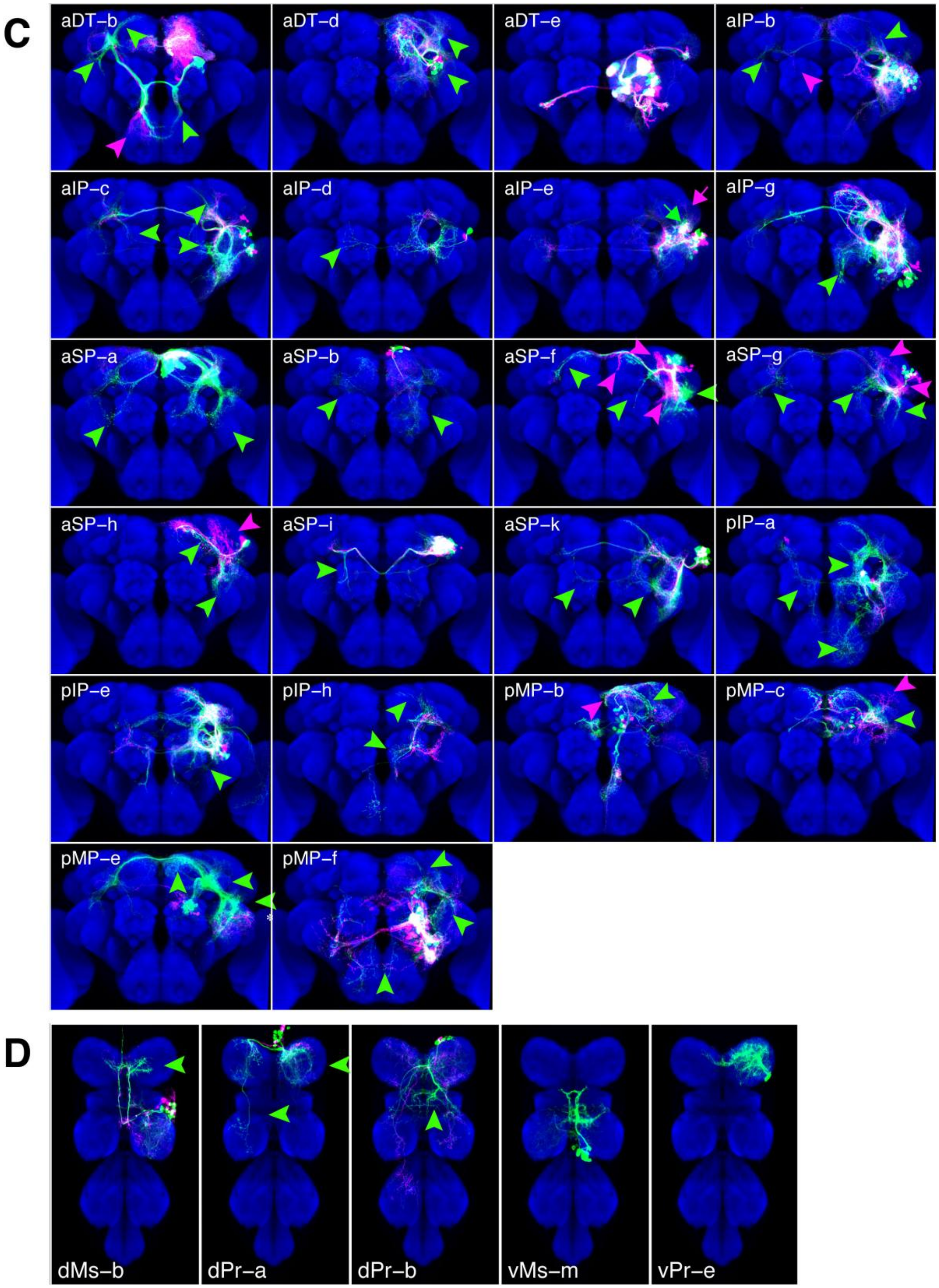
C
aDT–b
aDT–d
aDT–e
aIP–b
aIP–c
aIP–d
aIP–e
aIP–g
aSP–a
aSP–b
aSP–f
aSP–g
aSP–h
aSP–i
aSP–k
pIP–a
pIP–e
pIP–h
pMP–b
pMP–c
pMP–e
pMP–f
D
dMs–b
dPr–a
dPr–b
vMs–m
vPr–e

Figure S2. Comprehensive Atlas of *fru*+ Clones in the Central Nervous System (See Figure 2)

(A) Non-dimorphic clones expressing fru^{Gal4} in the central brain and suboesophageal ganglion. One example of each clone is show in a standard deviation z-projection overlaid on a similarly projected intersex template brain stained with nc82. Clones were extracted from raw stacks using a mask and the contrast has been increased in order to facilitate viewing of the fine processes of each clone. All clones shown are male.

(B) Non-dimorphic clones expressing fru^{Gal4} in the VNC. Images were processed as in (A).

(C) Comprehensive description of sexually dimorphic neuroblast clones in the central brain. One male (green) and one female (magenta) clone have been overlaid to demonstrate sexually dimorphic projections. For each clone the male and female image were extracted using an identical binary mask, a background subtraction and contrast adjustment (see methods) were used to make them comparable. White indicates strong overlap. Green and magenta arrowheads indicate male and female specific process respectively. Arrows indicate sex-specific arbours which are not evident in the image.

(D) Description of sexually dimorphic neuroblast clones in the VNC. Images are processed as per (C).

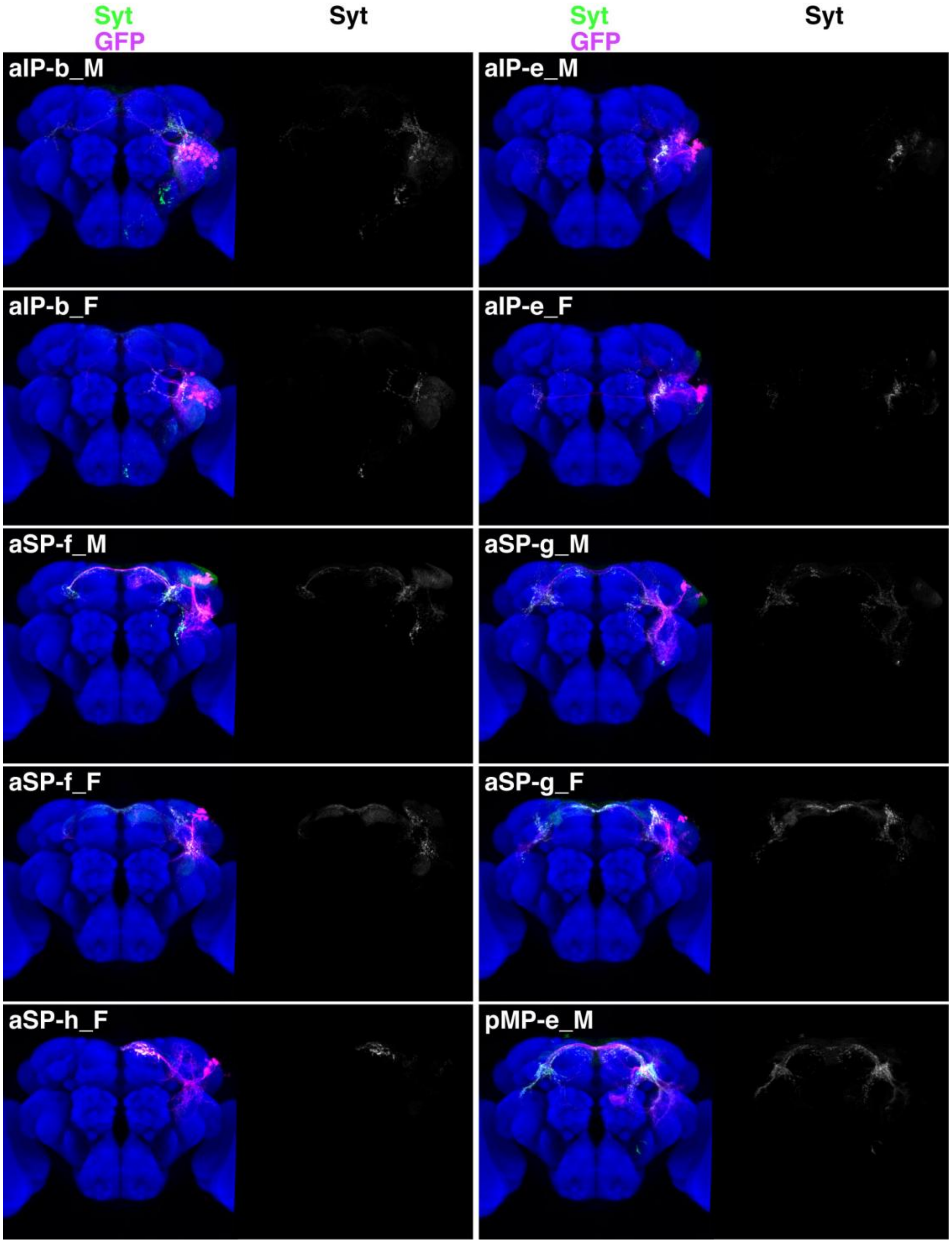

Figure S3. Synaptotagmin-HA Staining in *fru*+ Clones (See Figure 5)
Synaptotagmin-HA staining of fruGal4 clones. Images are registered to our intersex template. The left image in each panel is the Syt-HA image overlaid on the membrane bound GFP and the intersex template. The right image has only the Syt-HA staining for reference. The GFP and Syt-HA images have been background subtracted and the levels have been modified for demonstration purposes. Clone aSP-h only has Syt-HA data for females and pMP-e only has Syt-HA data for males in our dataset.

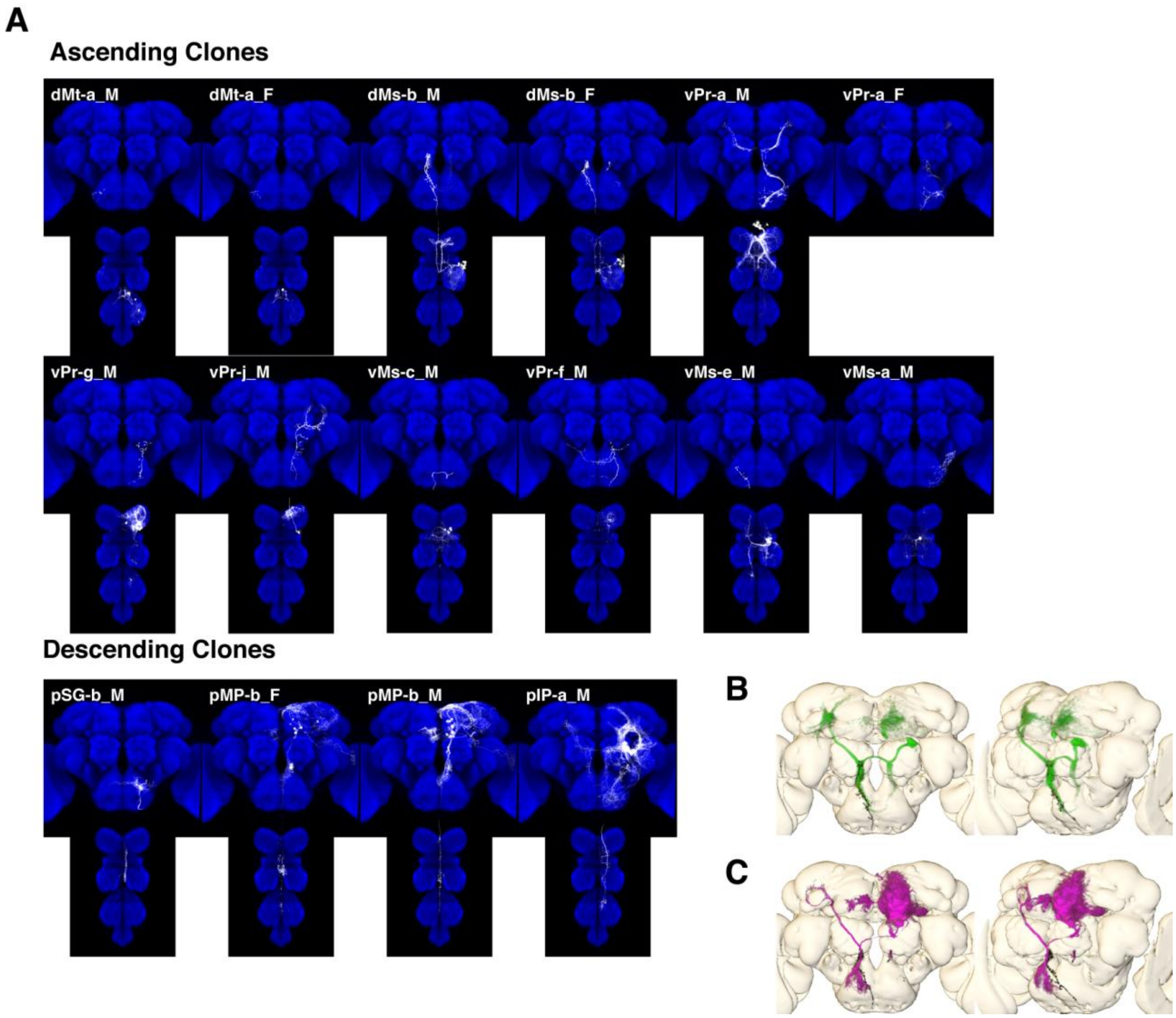

Figure S4. Ascending and Descending Processes (See Figure 4)
(A) One example of each clone is show in a standard deviation z-projection overlaid on a similarly projected intersex template brain or VNC stained with nc82. Clones were extracted from raw stacks using a mask and the contrast has been increased in order to facilitate viewing of the fine processes of each clone. An extractable VNC clone of vPr-a_F was not obtained so the central brain with the ascending process is shown in isolation.
(B and C) Example of sexually dimorphic overlap between ascending VNC clone dMs-b and brain clone aDT-b. In males the contralateral ascending projection of dMs-b has substantial overlap with aDT-b in the SOG while in females the ascending process largely misses the sex-specific arbour of aDT-b. Views are as in Figure 1B.

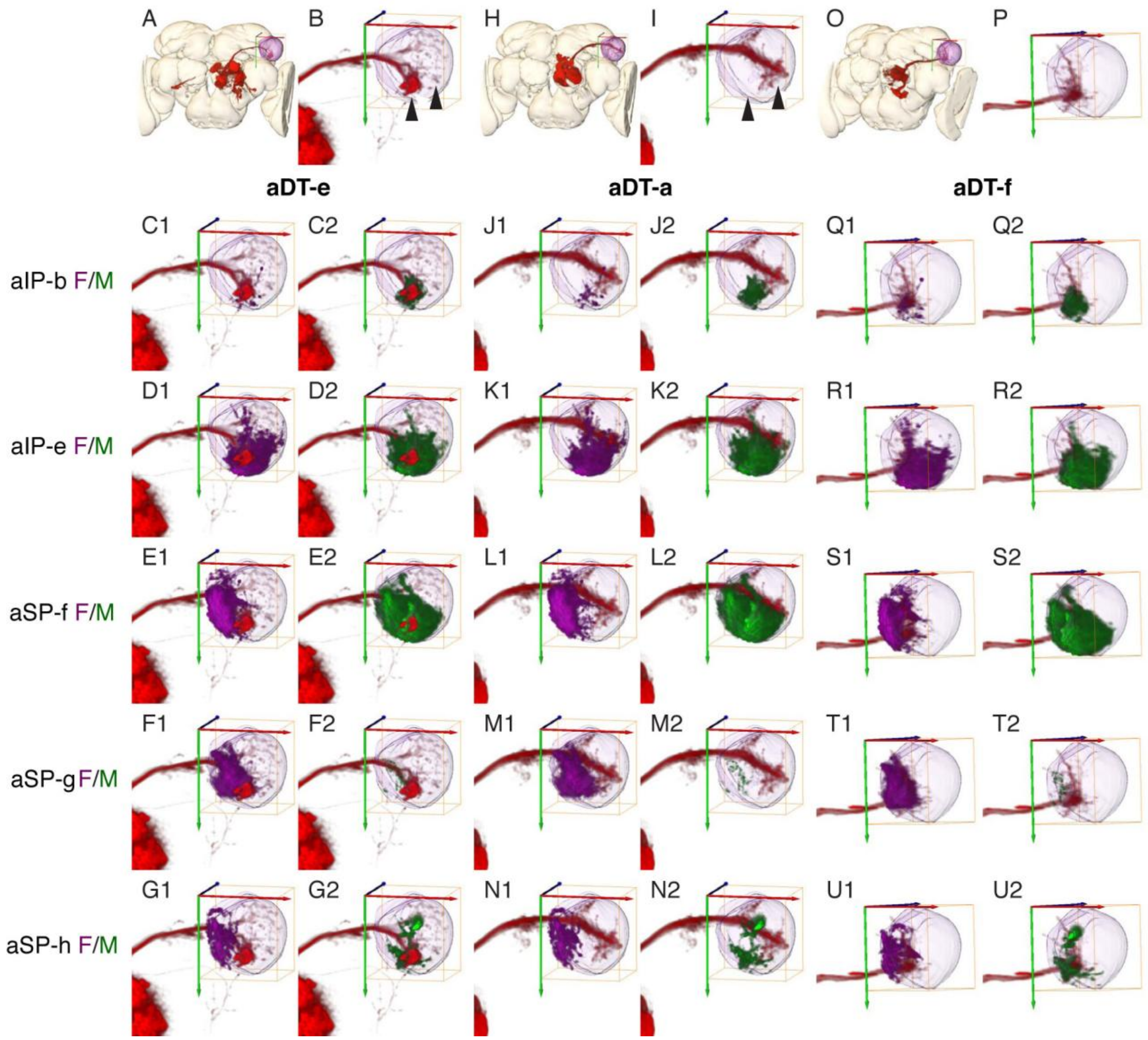

Figure S5. Potential Connectivity in Olfactory System (See Figure 2)
(A,B,H,I,O,P) Overview of aDT-e, a and f Projection Neuron clones. The lateral horn is shown as a purple surface surrounded by an orange bounding box. Arrows mark the mediolateral (red), dorsoventral (green) and anterior-posterior axis (blue) axes. Black arrowheads serve as reference for the positions of the main density of axon terminals of aDT-e (left arrowhead) and aDT-a (right arrowhead) in (B) and (I).
(C1—U2) Overlap between PN input (3 columns, red processes) and presumptive LHN output neurons (5 rows). For each LHN class, panels are shown with female (magenta) or male (green) clones, all of which are dimorphic.

Clone Name	Segment Information
**Ascending Clones**	
dMs-b	intersegmental
dMt-a	intrasegmental (crosses midline)
vMs-a	intersegmental
vMs-c	intersegmental
vMs-h	intrasegmental
vPr-a	intersegmental
vPr-f	intersegmental
vPr-j	intrasegmental (crosses midline)
**Motor Clones**	
dPr-e	intrasegmental
vMs-f	intrasegmental (crosses midline)
vMs-j	intersegmental
vMt-a	intrasegmental (crosses midline)
vPr-c	intrasegmental (crosses midline)
**Prothoracic Clones**	
SN-f	intrasegmental
dPr-a	intersegmental
dPr-b	intersegmental
dPr-c	intrasegmental (crosses midline)
dPr-d	intersegmental
dPr-e	intrasegmental
vPr-a	intersegmental
vPr-b	intersegmental
vPr-c	intrasegmental (crosses midline)
vPr-d	intrasegmental (paired)
vPr-e	intrasegmental (crosses midline)
vPr-f	intersegmental
vPr-g	intersegmental
vPr-h	intersegmental
vPr-i	intrasegmental
vPr-j	intrasegmental (crosses midline)
vPr-k	intrasegmental
vPr-l	intersegmental

Clone Name	Segment Information
**Mesothoracic Clones**	
SN-g	intrasegmental
dMs-a	intersegmental
dMs-b	intersegmental
dMs-c	intrasegmental (crosses midline)
vMs-a	intersegmental
vMs-b	intrasegmental (paired)
vMs-c	intersegmental
vMs-d	intrasegmental
vMs-e	intersegmental
vMs-f	intrasegmental (crosses midline)
vMs-g	intrasegmental (paired?)
vMs-h	intrasegmental
vMs-i	intrasegmental
vMs-j	intersegmental
vMs-k	intrasegmental
vMs-l	intrasegmental
vMs-m	intersegmental
vMs-n	intersegmental
vMs-o	intersegmental
**Metathoracic Clones**	
SN-h	intrasegmental
dMt-a	intrasegmental (crosses midline)
dMt-b	intersegmental
dMt-d	intrasegmental (crosses midline)
vMt-a	intrasegmental (crosses midline)
vMt-b	intrasegmental
vMt-c	intrasegmental (paired)
vMt-d	intrasegmental
vMt-e	intersegmental
vMt-f	intersegmental
vMt-g	intrasegmental (paired)
vMt-h	intrasegmental
vMt-i	intersegmental
**Abdominal Clones**	
dAb-a	intersegmental

Table S1. VNC Summary Table (See Table 1)

Clonal analysis of *fru*+ neurons in the VNC. Names and location of *fru*+ clones in the VNC.

Cachero et al MARCM Clones		Yu et al 2010	Kimura et al 2008 MARCM Clones			Lee et al 2000 Cluster
**Clone Name**	**Cell Number**	**Cluster Name**	**Clone Name**	**Largest Male Clone**	**Largest Female Clone**	**Fru Cluster**
aDT-a	32.75					
aDT-b	58.6 (28.6)	aDT2	mAL	27	5	fru-mAL
aDT-c	1		AL2	1	NA	fru-AL
aDT-d	25.2		AL1	17	NA	fru-AL
aDT-e	29	aDT3?	AL3	7	7	fru-AL
aDT-f	21					
aDT-g	22.4	aDT5				
aDT-h	51	aDT6	mcALa	18	14	fru-mcAL
aIP-a	22.67					
aIP-b	32.5 (19.6)					
aIP-c	30.8 (21.4)	aIP1	AL5b+AL6b	12	8	fru-AL
aIP-d	1					
aIP-e	27	aSP9	AL6a	14	14	fru-AL
aIP-f	23	pMP7?				
aIP-g	41.8333	pIP6				
aIP-h	11					
aSG-a	10.5					fru-SG
aSP-a	53 (22.6)	aSP2	aSP2	38	16	fru-aSP2
aSP-b	15.8(10.2)	aSP1?	aSP1	6	4	fru-aSP1
aSP-c	3	aSP1?				
aSP-d	48.25	aSP13	AL4	12	11	fru-AL
aSP-e	1					
aSP-f	23.2 (18.6)	aSP5	aSP3a	NA	NA	fru-aSP3
aSP-g	13.4	aSP6	aSP3b2	18	NA	fru-aSP3
aSP-h	5					
aSP-i	32.5	aSP11	AL5a	7	10	fru-AL
aSP-j	25.8	aSP12?				
aSP-k	29.2 (20.2)	aSP8	aSP3c	12	NA	fru-aSP3
aSP-l	24					
aSP-m	6					
Lo	NA	LP1	Lo	33	NA	fru- Lo
M	NA	M1	M			fru-M
pIP-a	14.33 (6.4†)	pIP1	P2b	14	NA	fru-P
pIP-b	24	pIP8	Lv2	10	4	fru-Lv
pIP-c	13.75	pIP3?/pIP7?				
pIP-d	NA		Lv1+Ld	45	28	fru-Ld+fru-Lv
pIP-e	34	pIP5/6	P4b	10	11	fru-P
pIP-f	1					
pIP-g						
pIP-h	1		P2a	1	1	fru-P
pIP-i	1					
pIP-j						
pMP-a	33.5 (23.2†)	pMP5?	pSP2-2	15	11	fru-pSP2
pMP-b	22	pMP5?	pSP1a (pSP1 in table S1, Kimura et al 2008)	4	NA	fru-pSP1
pMP-c		pMP1?				
pMP-d	1					
pMP-e	38.4	pMP4	P1	24	None	fru-P
pMP-f	21.33		P2e	4	2	fru-P
pMP-g	1					
pSG-a	5.5					fru-SG
pSG-b	12					fru-SG
pSG-c	NA					fru-SG
pSG-d	8					fru-SG
pSG-e	1					fru-SG
pSP-a	6.4					
pSP-b	31 (19)	pSP1?				
pSP-c						
pSP-d			aSP3b1	4	7	fru-aSP3
pSP-e	2.5					
pSP-f	2	pSP3?				
SN-e						

Table S2. Correspondence between *fruitless* Clusters from Different Studies (See Table 1)
Correspondences between this manuscript [4, 8] and Yu *et al.* (companion manuscript).

Supplemental Data on the Web

Upon publication of the manuscript, the following supplementary data will be made available. This will be at http://flybrain.stanford.edu unless another location is indicated.

- Interactive version of Figure 4 available at http://flybrain.mrc-lmb.cam.ac.uk/Cachero/InteractiveHeatmap/DimorphicRegionsOverlapMale.html
- Confocal stack double stained for fruGal4 driven membrane targeted GFP and anti Doublesex antibody
- Amira scripts allowing interactive volume rendering of arbitrary groups of *fru+* clones. Different neurons can be immediately (un)selected for display with checkboxes and a button allows switching between display of male and female clones where these are dimorphic.
- At least one masked stack of every clone
- ... including male and female examples where these are dimorphic
- Masks for each clone
- Average male, female and intersex templates
- Registration files used for DBM
- Simple image registration command line and GUI control tools (open source)
- Source code for Image Analysis Tools in R (open source)
- ImageJ plugins for Biorad PIC (w) and NRRD (r/w) IO (open source with Fiji)
- Amira plugins for NRRD IO (open source)
- We will also copy all raw and processed image data for our study onto a 1.5TB external hard drive on request (LSM confocal files, registration files, registered images). These raw data are freely available for study by our colleagues. We would however request that 1) any future studies that analyse our data in new ways, freely release the derived data and 2) any future studies that combine our data with other new data should release those new data on a similar basis upon publication.

Supplemental Experimental Procedures

Image Registration

We used nc82 neuropil staining from multichannel confocal stacks as the input to a fully automatic intensity-based (landmark free) 3D image registration software [9, 10]. An initial linear registration with 9 degrees of freedom (translation, rotation and scaling of each axis) was followed by a non-rigid registration that allows different brain regions to move somewhat independently, subject to a smoothness penalty [11]. The underlying deformation model is based on third order B splines; for the central brain the final grid of B spline control points was 51 x 51 x 35, for a total of 91035 control points and therefore 273105 degrees of freedom. The algorithm typically held about two thirds of the control points fixed because they contained no useful information ˜ usually locations outside the brain. The final control point grid had a mean spacing of 5 to 6 µm. The registration for the nc82 channel could then be applied to other channels containing labelled neurons.

We used an updated version of the core registration software which we have made available at http://flybrain.stanford.edu along with a control script used to coordinate multiple registrations. In the later stages of the project, the core registration toolkit (CMTK) was made open source and is now available at http://www.nitrc.org/projects/cmtk/. Registrations were run either on an Apple Mac Pro (8 core) or on a Sun Gridengine 6.2 Cluster, consisting of 33 IBM 8 core Xeon blades, running Centos 5.2 Linux; registration jobs were run on 4 cores and took 1–4h to complete. The only pre-processing of raw confocal images before registration was rotation to match the orientation of the template to the nearest 90 degrees, followed by export in Biorad PIC format using a plugin that we have contributed to the Fiji distribution of ImageJ (http://pacific.mpi-cbg.de). We have also bundled plugins to read and write registration output in the NRRD image format (http://teem.sourceforge.net/nrrd/format.html) with Fiji.

Deformation-Based Morphometry

Deformation-Based Morphometry (DBM) is a sensitive and global approach to identifying structural differences. The basic approach is to look for systematic differences in the amount of expansion or contraction required to map each point in a sample brain onto the template. The relative volume change is measured by the Jacobian determinant; this has a value of 1 when there is no change in volume, with values above and below 1 indicating expansion and shrinkage, respectively. Before analysis the raw Jacobian determinants were first normalised to remove the effect of overall brain size by dividing by the mean Jacobian determinant for the whole brain [10].

We then carried out two types of analysis. The first was voxel-wise, generating a t-statistic parametric map for the difference in volume between two groups. For each voxel in the template brain, we calculated the normalised Jacobian determinants for the registration of each sample brain, log transformed those determinants [12], and then carried out a t-test. For example, if there were two groups of 40 sample brains each voxel in the resulting parametric map would contain a t-statistic resulting from an unpaired test with 78 degrees of freedom. The measurements were carried out on a grid of 2 x 2 x 2 µm voxels, but only inside the neuropil, a total of 606729 voxels. We used permutation testing [13] to estimate a null distribution for the t-statistic. To achieve a one tailed alpha of 0.05 for female and male enlarged regions across the whole neuropil we calculated the 5% and 95% quantiles, respectively, for the extreme values of 5000 permutations. This gave t values of -4.96 and 5.00, respectively. For simplicity we rounded these to $t=\pm5$. For comparison, the extreme t values for all 5000 permutations were -7.32 and 6.48.

We also carried out volumetric measurements by summing the normalised Jacobian determinants over a region of interest and then multiplying by the voxel size; this is equivalent to defining a region of interest (ROI) in template space, reverse transforming that ROI into the space of each sample brain and then measuring the volume of the transformed ROI in that sample brain.

We noticed two practical issues in the statistical analysis. The first was that voxel-wise DBM across the whole brain appears sufficiently sensitive to pick up structural variations that are likely to be due to genetic background. The second issue was that when carrying out DBM-based volumetric analysis, there were significant batch effects (in spite of the normalisation by total brain volume). This could be due to differences in antibody penetration or brain shrinkage during

immunochemistry. Such batch differences would tend to reduce the effective number of degrees of freedom for any statistical test leading to false positives, so we therefore modelled them explicitly with a mixed effects model using the nlme package of R [14, 15].

Masking of Registered Clones

In order to analyse individual clones from brains with high levels of superficial background fluorescence or additional non-overlapping clones, masks were generated with Fiji's Segmentation Editor plugin. Three registered female image stacks containing the same clone were selected and merged to a single RGB image. A paint brush tool was used to select regions that contained shared processes and these shared regions were exported as a binary mask. This image file was overlaid with a male stack having the corresponding clone and evaluated to determine if the mask needed expansion for male clones. If the mask needed expansion, three registered male brains expressing the clone of interest were then overlaid, opened in the Segmentation Editor and the 8-bit female mask was added to the plugin's label field using the Image Calculator in Fiji; note that the mask was never made smaller, as a result, the male mask functioned as a logical OR of the female and male patterns of the clone. This new mask was exported, saved and compared to a male stack to see if the mask covered all of the processes in a registered image. As MARCM clones are unilateral, all masks were made with the assumption that the cell bodies of the clone would be located on the left side of the fly's brain (the image's right side).

To extract clones from raw images, the mask file was multiplied with a registered stack containing the clone of interest using the Image Calculator in Fiji or by passing the mask file to the CMTK reformatx tool. If the clone of interest was on the fly's right, the image was first horizontally flipped and then further transformed using a bridging registration of the template brain to its horizontally flipped self. The end result was a registered image stack containing the clone of interest without any adjustments to voxels inside the masked region, but with all voxels outside the masked region set to zero.

Image Processing and Visualisation

In order to prepare images for further analysis or display, two additional processing steps were carried out. The anti-GFP/CD8 antibodies resulted in an appreciable level of background staining within the neuropil. We fitted a Gaussian distribution to these background voxel intensities and subtracted a threshold value of □ + 2□ from all voxels. We then mapped the remaining intensities onto an 8 bit range from 0 to 255 using the unu **quantize** tool (http://teem.sourceforge.net/unrrdu). For overlap analysis, we downsampled images fivefold in xy and twofold in z, first applying a Gaussian convolution with □ = 1.0 to eliminate spatial frequencies above the new voxel size (2.06 x 2.06 x 2.14 µm).

For visualisation in Amira 5.2.2 (www.amiravis.com) images were cropped to the smallest cube containing all non-zero voxels using the CMTK convert tool; this reduced the image size by an average of >80% easing visualisation. Some visualisations depended on label fields to segment the arbours of a particular neuroblast clone into specific zones. Label fields were constructed in Amira using the smoothed images mentioned above and a combination of thresholding and the

lasso tool in the 3D viewer. Label fields were then used in combination with the Amira Volren module for visualisation or used to multiply the original image data for overlap analysis. The same procedure was used to isolate just the dimorphic arbours from the 21 dimorphic clones in the central brain. The volume of the dimorphic arbours defined by these label fields is a reasonable approximation of the volume of neuropil that could be sampled by the processes of each clone.

Confocal images of twin-spot MARCM brains were deconvolved with Huygens Essential (Scientific Volume Imaging, Hilversum, The Netherlands) using the classic MLE algorithm, a theoretical point spread function that corrects for spherical aberration due to sample depth and assuming a signal to noise ratio of 20. The images were then opened in Fiji and the brightness and contrast were adjusted and regions of interested were selected and expanded for display purposes. The blue channel in Figure 6C—F was calculated as follows: First, green and magenta channels were manually thresholded. Second, voxels were set to blue if they were positive in both channels or if they were positive in one channel and a voxel within a cube of edge 3 centred in the voxel was positive in the other channel (i.e. contiguous pixels).

For display purposes, the full *fru*^{Gal4} expression pattern was averaged between two male (m) and two female (n) brains. Two brains of each sex were registered to our intersex template and averaged using Fiji. In addition, two masks have been applied to the averaged brains, one to remove the cell bodies on the surface of the brain and a second to remove the mushroom bodies. The RGB Rainbow lookup table from Fiji was used to enhance contrast.

Cluster and Overlap Analysis

Cluster analysis in Figure 5 was carried out using the R function **hclust** using Ward's method which aims to find compact, spherical clusters. The input was a distance matrix calculated using the **dist** function from Pearson cross-correlation scores of all raw LHN images cropped to a cube surrounding the lateral horn.

Overlap analysis of olfactory clones in Figure 6 was carried out using data that retained intensity information rather than using binary masks. Quantile based normalisation was first applied to all of the images of a particular clone and sex using the **normalize.quantiles** function of R package **affy**. This compensated for arbitrary differences in the intensity of different images of the same clone due to differences in staining intensity or confocal microscope acquisition parameters. We then calculated the dot product of each combination of PN input clone and LHN output clone.

Supplemental References

1. Rideout E.J., Billeter J.C., and Goodwin S.F. (2007). The sex-determination genes fruitless and doublesex specify a neural substrate required for courtship song. Curr. Biol. *17* (17), 1473–8.
2. Villella A. and Hall J.C. (2008). Neurogenetics of courtship and mating in Drosophila. Adv. Genet. *62*, 67–184.
3. Mellert D.J., Knapp J.M., Manoli D.S., Meissner G.W., and Baker B.S. (2010). Midline crossing by gustatory receptor neuron axons is regulated by fruitless, doublesex and the Roundabout receptors. Development *137* (2), 323–32.
4. Lee G., Foss M., Goodwin S.F., Carlo T., Taylor B.J., and Hall J.C. (2000). Spatial, temporal, and sexually dimorphic expression patterns of the fruitless gene in the Drosophila central nervous system. J. Neurobiol. *43* (4), 404–26.
5. Yu J.Y., Kanai M., Demir E., Jefferis G.S.X.E., and Dickson B.J. (2010). Cellular organization of the neural circuit that drives Drosophila courtship behavior. Curr. Biol. *this issue*.
6. Otsuna H. and Ito K. (2006). Systematic analysis of the visual projection neurons of Drosophila melanogaster. I. Lobula-specific pathways. J. Comp. Neurol. *497* (6), 928–58.
7. Kimura K.I., Ote M., Tazawa T., and Yamamoto D. (2005). Fruitless specifies sexually dimorphic neural circuitry in the Drosophila brain. Nature *438* (7065), 229–233.
8. Kimura K.I., Hachiya T., Koganezawa M., Tazawa T., and Yamamoto D. (2008). Fruitless and doublesex coordinate to generate male-specific neurons that can initiate courtship. Neuron *59* (5), 759–769.
9. Rohlfing T. and Maurer C. R. J. (2003). Nonrigid image registration in shared-memory multiprocessor environments with application to brains, breasts, and bees. IEEE Trans. Inf. Technol. Biomed. *7* (1), 16–25.
10. Jefferis G.S.X.E., Potter C.J., Chan A.M., Marin E.C., Rohlfing T., Maurer C.R.J., and Luo L. (2007). Comprehensive maps of Drosophila higher olfactory centers: spatially segregated fruit and pheromone representation. Cell *128* (6), 1187–1203.
11. Rueckert D., Sonoda L.I., Hayes C., Hill D.L., Leach M.O., and Hawkes D.J. (1999). Nonrigid registration using free-form deformations: application to breast MR images. IEEE Trans. Med. Imaging *18* (8), 712–21.
12. Rohlfing T., Sullivan E.V., and Pfefferbaum A. (2006). Deformation-based brain morphometry to track the course of alcoholism: differences between intra-subject and inter-subject analysis. Psychiatry Res. *146* (2), 157–70.
13. Nichols T.E. and Holmes A.P. (2002). Nonparametric permutation tests for functional neuroimaging: a primer with examples. Hum. Brain Mapp. *15* (1), 1–25.
14. Pinheiro J.C. and Bates D.M. (2000). Mixed-effects models in S and S-PLUS (Springer-Verlag).
15. R Development Core Team (2009). R: A Language and Environment for Statistical Computing. R Foundation for Statistical Computing, Vienna, Austria. ISBN 3-900051-07-0.
